# Primary Role of Semi-Local and Exact-Exchange Difference in the Description of Correlation Effects in Hybrid Functionals

**DOI:** 10.1021/acs.jctc.6c00843

**Published:** 2026-09-21

**Authors:** Vignesh Balaji Kumar, Szymon Śmiga, Rodrigo Araújo Mendes, Bogumiła Jezierska, Rodney Bartlett, Ireneusz Grabowski

**Affiliations:** † Institute of Physics, Faculty of Physics, Astronomy, and Informatics, 317747 Nicolaus Copernicus University in Toruń, ul. Grudzia̧dzka 5, 87-100 Toruń, Poland; ‡ Institute of Advanced Studies, Nicolaus Copernicus University in Toruń, ul. Wileńska 4, 87-100 Toruń, Poland; § Quantum Theory Project, 3158 University of Florida, Gainesville, Florida 32611, United States

## Abstract

Hybrid density functional approximations are among the most widely used electronic-structure methods, yet the physical origin of their representation of correlation effects remains incompletely understood. In this work, we analyze correlation in hybrid functionals from the perspectives of Kohn–Sham exchange–correlation potentials, correlated densities, and energy-related observables. We show that the dominant qualitative features of correlation in hybrid DFAs do not arise primarily from the semilocal correlation functional itself, but instead from the difference between the semilocal exchange and exact-exchange contributions. This exchange-difference term is essential for restoring the physically correct structure of the correlation component of the exchange–correlation potential and the corresponding correlated density, whereas the semilocal correlation part acts mainly as a compensating contribution in the core and valence regions. Based on this analysis, we propose an interpretable construction principle for hybrid functionals in which the semilocal/exact-exchange difference is scaled in a controlled way. As a proof of concept, this methodology yields the simple global hybrid UMK26 functional. Despite its uncomplicated global-hybrid form, the functional produces correlation potentials and correlated densities in good agreement with CCSD­(T) references, delivers accurate ionization potentials from orbital energies, and yields very good charge-transfer excitation energies, performing on a comparable footing with several recently proposed range-separated hybrids. Although UMK26 is not uniformly optimal across all thermochemical benchmarks, it performs particularly well for self-interaction-sensitive systems and provides a physically transparent route for the rational design of future hybrid density functionals.

## Graphical abstract

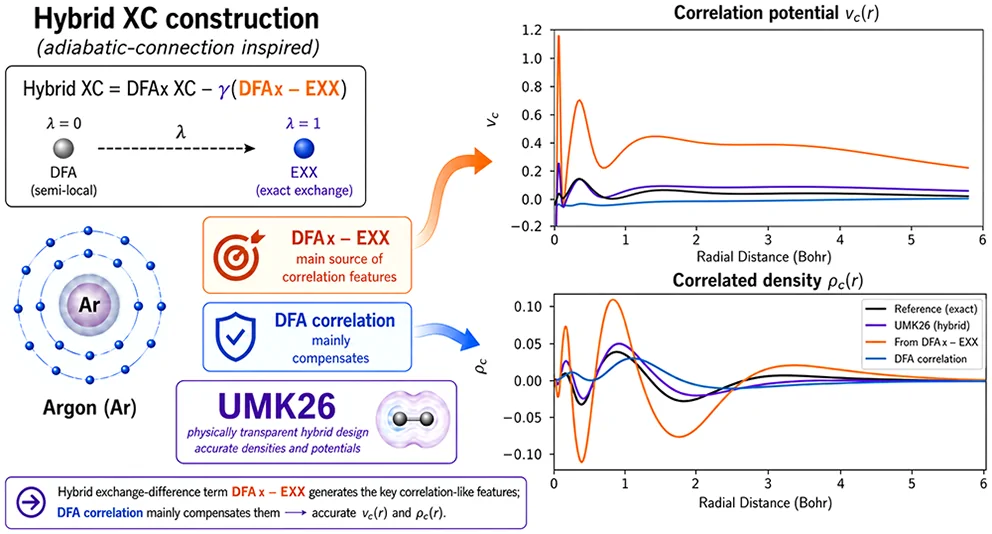

## 1 Introduction

The Kohn–Sham density functional theory (KS-DFT)^1^ is one of the most popular computational methods, offering a cheaper and faster alternative to traditional *ab initio* wave function approaches. The key element of this methodology is the exchange-correlation (XC) energy functional, which accounts for many-body effects beyond the Hartree approximation and the noninteracting kinetic energy. Of course, the correct formula for XC functionals remains unknown and will likely remain so, despite the enormous effort put into their development. However, to date, many practical classes of density functional approximations (DFAs) have been developed.^2,3^ Among them, the so-called hybrid (HYB) class of DFAs^4^ are common in standard KS-DFT applications, offering the best compromise between accuracy and efficiency.^5–10^ In general, HYB XC functionals (*E*_*xc*_^HYB^) are defined as a mixture of a fraction of the Hartree–Fock (HF) exact-exchange (EXX) energy (*E*_*x*_^EXX^) with additional amounts of semilocal exchange and correlation functionals, usually defined at LDA, GGA, and meta-GGA levels.^11^ Formally, they can be defined using the density-fixed adiabatic connection (AC) formalism.^4,12,13^ To recall, the AC XC functional definition is based on the coupling constant (α ≥ 0) integral formula^14–19^

1 $$E_{\mathrm{xc}}\left[\rho \right]=\int_{0}^{1}d\alpha W_{\mathrm{xc}}^{\alpha }\left[\rho \right]$$

with integrand defined as *W*_*xc*_^α^[ρ] = ⟨Ψ^α^[ρ]|*V̂*_*ee*_|Ψ^α^[ρ]⟩ – *U*[ρ], where *V̂*_*ee*_ is the Coulomb operator, *U*[ρ] is the Hartree energy, and Ψ^α^[ρ] is the α-dependent ground state electronic wave function, which yields for any value of α the density ρ­(**r**). eq 1 connects a noninteracting KS single particle determinant (α = 0) with a real, fully interacting one (α = 1). This provides the exact and formal definition of the XC functional. Two limiting cases of the *W*_*xc*_^α^[ρ] expansion are essential for the HYB DFA construction, namely the α → 0 and the full interacting limit at α → 1. Using those, one can construct the general approximate integrand formula, which defines the HYB class of DFAs

2 $$W_{\mathrm{xc},\alpha }^{\mathrm{HYB}}\left[\rho \right]=W_{\mathrm{xc},\alpha }^{\mathrm{DFA}}\left[\rho \right]-\left(E_{x}^{\mathrm{DFA}}\left[\rho \right]-E_{x}^{\mathrm{EXX}}\right){\left(1-\alpha \right)}^{\left(n-1\right)}$$

where *E*_*x*_^DFA^[ρ] is the semilocal DFA exchange energy, and *W*_*xc*,α_^DFA^[ρ] the semilocal DFA coupling-constant integrand derived from the Levy-Perdew scaling relation^20^

3 $$W_{\mathrm{xc},\alpha }^{\mathrm{DFA}}\left[\rho \right]=E_{x}^{\mathrm{DFA}}\left[\rho \right]+2E_{c}^{\mathrm{DFA}}\left[\rho_{1/\alpha }\right]\alpha +\frac{\partial E_{c}^{\mathrm{DFA}}\left[\rho_{1/\alpha }\right]}{\partial \alpha }\alpha^{2}$$

with ρ_1/α_(**r**) = α^–3^ρ­(**r**/α) being the coordinate-scaled^21,22^ density. Equation 2 correctly reduces to the *E*_*x*_^EXX^ energy expression when α = 0 and to *W*_*xc*,α_^DFA^[ρ] at α = 1, where this approximation is most trustworthy.^13^ The parameter *n* ∈ ⟨0, ∞) is the curvature parameter which controls the shape of the approximate AC integrand.

Using eq 1 and taking into account that close to the upper limit (α = 1) eq 3 can be approximated by *W*_*xc*_^1^[ρ] ≈ *E*_*x*_^DFA^[ρ] + 2*E*_*c*_^DFA^[ρ], one obtains^13^

4 $$E_{\mathrm{xc}}^{\mathrm{HYB}}\left[\rho \right]=E_{\mathrm{xc}}^{\mathrm{DFA}}\left[\rho \right]-\frac{1}{n}\left(E_{x}^{\mathrm{DFA}}\left[\rho \right]-E_{x}^{\mathrm{EXX}}\right)$$

which gives the general HYB XC energy expression, where the 1/*n* factor controls the semilocal and HF-exchange difference contributions in the HYB DFA. For instance, PBE0,^23^ with 25% exact exchange, corresponds to *n* = 4, while B3LYP,^24^ with 20% exact exchange, corresponds to *n* = 5. The role of the exact-exchange mixing coefficient has recently been explored in the context of machine-learning (ML) assisted HYB DFA construction^25^ showing that the adaptive system-dependent exact-exchange fraction can lead to improved quality of HYB DFA preditions.

Conceptually, this is illustrated in Figure 1 where the gray area corresponds to the AC XC functional eq 1, whereas the orange dashed one represents the HYB XC energy defined via eq 4. It is important to stress that the exchange energy difference term appearing in eq 4 is not system-specific, but follows directly from the AC construction of hybrid functionals.

**Figure 1:**
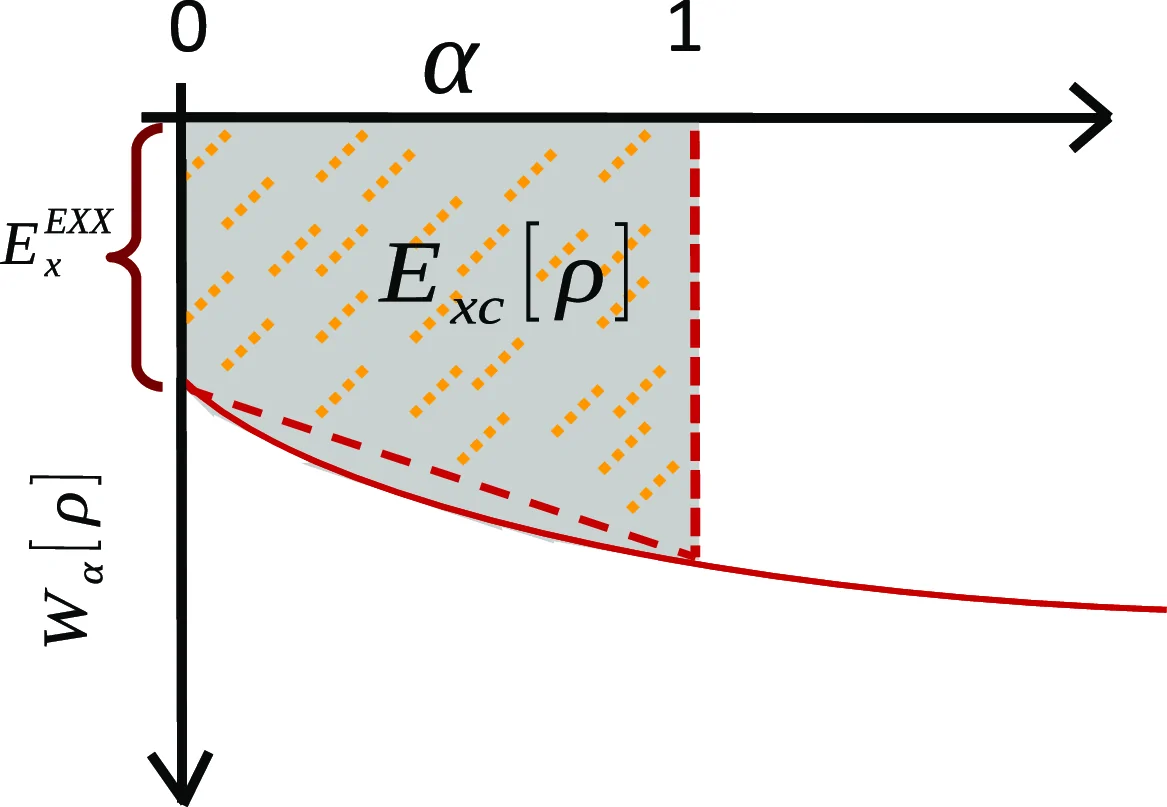
Graphical representation of the adiabatic connection formula.^18^ The gray area corresponds to AC XC energy (see eq 1). The red dashed line represents the approximated integrand formula given by eq 2, whereas the orange dashed area represents the HYB DFA XC energy defined by eq 4.

Although the HYB DFAs are developed solely within the KS framework, their practical implementation is performed within the generalized KS (GKS) framework,^26–28^ where the XC potential remains nonlocal. This is due to the orbital dependence of the nonlocal HF exact-exchange energy term. This is also one of the reasons why the KS HYB XC potentials are not typically investigated during functional development. We recall, however, that these local XC potentials are well-defined quantities entering the KS equations directly.^29,30^ In addition to many other necessary features, an XC functional approximation should exhibit an accurate description of the KS XC potential. The good performance of an XC functional for some property, e.g., the total energy, without a correct XC potential, is artificial. Applications of such XC approximations are subject to numerous practical if-else conditions, which are among the present and long-term challenges faced by DFT.^17,31–33^

As shown in a recent study,^29,30,34^ hybrid functionals can provide reasonably accurate local XC potentials within the KS scheme via inverse procedures.^29^ Moreover, it was shown^30,34^ that the correlation functionals (*E*_*c*_^HYB^), which can be defined for any HYB XC approximation as

5 $$E_{c}^{\mathrm{HYB}}\left[\rho \right]=E_{\mathrm{xc}}^{\mathrm{HYB}}\left[\rho \right]-E_{x}^{\mathrm{EXX}}$$

give very physical correlation potentials, capable of correctly describing the quantum shell oscillations in the high-level reference data for atoms and molecules, unlike other semilocal functionals.^35,36^ Thus, we have recently developed a critical methodology for evaluating the quality of HYB XC DFA, where the KS XC potentials, together with other quantities,^30^ serve as a comprehensive tool to assess the quality of existing and new HYB approximations.

We also note that the local XC potential *v*_xc_ in DFT can be viewed as a local counterpart of the optical potential, namely, the self-energy Σ­(*E*) entering the Dyson equation for the one-particle Green’s function.^37,38^ Unlike *v*_xc_, Σ­(*E*) is generally nonlocal and energy dependent. The two descriptions are formally connected through the Sham–Schlüter equation,^39^ which determines a local potential reproducing the Green’s-function density.^38^ In the correlated-orbital formulation discussed by Bartlett,^40^ incorporating coupled-cluster theory, removes the explicit frequency dependence and leads to the IP- and EA-EOM-CC one-particle picture. Projection of this correlated-orbital theory onto DFT yields QTP functionals satisfying the corresponding correlated-orbital conditions.^40,41^ The present work concerns the static local KS/GKS representation of correlation and does not calculate the full energy-dependent self-energy.

In the present study, we examine how correlation effects are represented in the XC, exchange-only, and semilocal correlation parts of the KS potentials and energies within the HYB class of functionals, and how these representations affect other key quantities, including correlated densities and ionization potentials (IPs). We show that the semilocal and HF-like exchange-energy difference terms appearing in eqs 2 and 4 play a central role in accurately describing correlation effects in HYB functionals, at both the potential and electron-density levels.

Building on this insight, we then propose a methodology for the interpretable construction of XC DFAs, which we use to develop a global hybrid XC functional that performs remarkably well for the IPs and charge transfer (CT) excitation energies of a wide range of species.

## 2 Methogology

We start from reformulating eq 4 into the following form

6 $$E_{\mathrm{xc}}^{\mathrm{HYB}}\left[\rho \right]=E_{x}^{\mathrm{EXX}}+\frac{n-1}{n}\left(E_{x}^{\mathrm{DFA}}\left[\rho \right]-E_{x}^{\mathrm{EXX}}\right)+E_{c}^{\mathrm{DFA}}\left[\rho \right]$$

The above representation, together with eq 5, allows the correlation HYB functional to be written as

7 $$E_{c}^{\mathrm{HYB}}\left[\rho \right]=\frac{n-1}{n}\left(E_{x}^{\mathrm{DFA}}\left[\rho \right]-E_{x}^{\mathrm{EXX}}\right)+E_{c}^{\mathrm{DFA}}\left[\rho \right]=E_{c}^{\mathrm{XDiff}}\left[\rho \right]+E_{c}^{\mathrm{DFA}}\left[\rho \right]$$

where *E*_*c*_^XDiff^[ρ] explicitly stands for the difference of the exchange-only DFA functional and HF-like one, scaled by (*n* – 1)/*n* factor. For a global hybrid, the fraction of exact exchange (*a*_EXX_) is connected with the scaling prefactor of the exchange-difference contribution as (*n* – 1) /*n* = 1 – *a*_EXX_. Thus, increasing the amount of exact exchange directly reduces the *E*_*c*_^XDiff^ contribution, which vanishes in the limit of 100% exact exchange. A similar construction can also be applied to the range-separated^42,43^ (RS) hybrid DFAs.

In the present study, we can consider two key cases of eq 6:

- for the *n* ≠ 1 case, we see that the HYB correlation functional *E*_*c*_^HYB^[ρ] consists of two separable contributions: one, which depends solely on exchange-only terms (*E*_*c*_^XDiff^[ρ]), and the standard semilocal correlation functional (*E*_*c*_^DFA^[ρ]).
- for *n* = 1, eq 6 reduces to *E*_*xc*_^HYB^[ρ] = *E*_*x*_^EXX^ + *E*_*c*_^DFA^[ρ] which incorporates 100% of EXX energy, whereas the correlation term is represented solely by the semilocal approximation (*E*_*c*_^DFA^[ρ]). In some cases, this construction can outperform the HF/EXX method’s predictions.^44–46^ However, from a broader perspective, it suffers from a lack of compatibility between exact-exchange and semilocal correlation. This aspect will be discussed in more detail later.

In both cases, we investigate the effects of electron correlation by examining KS correlation potentials and correlated densities calculated for several HYB DFAs. The correlated density is defined as^35,36,47^

8 $$\rho_{c}\left(r\right)=\rho^{\mathrm{method}}\left(r\right)-\rho^{\mathrm{xref}}\left(r\right)$$

where ρ^xref^ is the density obtained from the exact-exchange (xref = EXX) and HF (xref = HF) calculations for KS-DFT and conventional WFT methods, respectively. In the case of atoms, we consider difference radial-density (DRD)^35,48^ distributions defined as *DRD*(*r*) = 4*π r* ^2^ρ_*c*_(*r*). In turn, correlation potentials in our considerations are defined as

9 $$v_{c}\left(r\right)=v_{\mathrm{xc}}^{\mathrm{method}}\left(r\right)\left[\rho^{\mathrm{method}}\right]-v_{x}^{\mathrm{HF}}\left(r\right)\left[\rho^{\mathrm{HF}}\right]$$

i.e., the difference between the XC potential obtained for a given method and that obtained from HF density. Both potentials are computed using the Wu-Yang (WY)^49^ inverse method. All calculations have been performed using an identical computational setup as in ref 30. for the (coupled cluster singles-doubles with perturbative triples) CCSD­(T)^50^ benchmark set.

We note that full analytical analysis of the corresponding local potentials is highly challenging, because the exact-exchange contribution is orbital-dependent and the associated local KS potential must be obtained through the optimized-effective-potential formalism or by inverse procedures such as the WY method. The present numerical analysis, therefore, relies on high-quality reference densities and accurate inversion techniques, while the formal origin of the exchange-difference in eq 7 follows directly from the hybrid AC expression.

## 3 Results

We begin our considerations with the construction for *n* ≠ 1 in eq 6. To this end, we have assessed four commonly applied global HYB DFAs, namely PBE0,^23^ B3LYP,^24^ QTP17,^51^ and one RS HYB LC-ωPBE^52^ variant. First, we turn our attention to the correlation energies calculated using eq 7, which usually provide more accurate results than those obtained from the semilocal DFA formula. This trend is illustrated in Table 1, where we report the correlation energies for several systems computed using the representative PBE0 hybrid DFA. As can be observed, the semilocal component typically overestimates the correlation energy (MAE = 0.090 E_*h*_, MARE = 20.32%). In contrast, the *E*_*c*_^XDiff^[ρ] contribution, generally positive in value, mitigates this overestimation, resulting in improved hybrid correlation energies (MAE = 0.065 E_*h*_, MARE = 18.49%). Therefore, this term should not be interpreted as an uncontrolled double counting of correlation, but rather as a compensating contribution. Similar trends are observed for the other two functionals tested, i.e., B3LYP and LC-ωPBE, reported in the Supporting Information (SI) (see Tables S2–S3).

**Table 1:** Correlation Energies (in E_*h*_) for Several Systems Computed for PBE0 HYB Functional Using Eq 7 (Third Column) and PBE Semi-Local Correlation Energy Expression (Fourth Column) using Self-Consistent PBE0 Reference^a^

systems	CCSD(T)	eq 7	*E*_*c*_^PBE^[ρ]	*E*_*c*_^XDiff^[ρ]
*He*	–0.0411819	–0.0341513	–0.041400	0.0072487
*H*_2_	–0.0404878	–0.0358609	–0.0432167	0.0073558
*Be*	–0.088351	–0.0649265	–0.0856687	0.0207422
*He*_2_	–0.082365	–0.0683034	–0.0827999	0.0144965
*Ne*	–0.3621118	–0.3295189	–0.3484019	0.018883
*H*_2_O	–0.3509719	–0.3253928	–0.3260439	0.000651
*HF*	–0.3644942	–0.3359948	–0.3363827	0.000388
*NH*_3_	–0.3222876	–0.3004125	–0.3154617	0.0150492
*Mg*	–0.2189615	–0.3607493	–0.4098516	0.0491023
*CO*	–0.5025411	–0.4583556	–0.4494995	–0.008856
*N*_2_	–0.5173779	–0.4699707	–0.4525074	–0.017463
*Ar*	–0.4433619	–0.5764346	–0.7055727	0.1291381
*C*_2_H_6_	–0.534443	–0.5040304	–0.5605092	0.056479
*HCl*	–0.4369698	–0.5748036	–0.6873607	0.1125571
*Ne*_2_	–0.7244349	–0.6593054	–0.6968715	0.037566
*Cl*_2_	–0.8398284	–1.1247353	–1.3285813	0.203846
MAE		0.065	0.090	
MARE[%]		18.49	20.32	

a The last column presents the scaled energy difference. The MAE (in E_*h*_) and MARE are calculated with respect to CCSD­(T) reference data (second column). All calculations have been performed in the uncontracted aug-cc-pVQZ basis set.

Next, in Figure 2, we report the correlation potentials and correlated densities of the Ar atom, obtained with and without the semilocal correlation functional *E*_*c*_^DFA^[ρ] in eq 6, presented in the top and bottom panels, respectively. Similar plots for a few other systems (Be, Ne, CO, and H_2_O) are shown in the SI file (see Figures S1–S4). Inspecting the fully correlated case (top panel) in Figure 2, one can note that all the investigated functionals yield correlation potentials that are qualitatively comparable to the CCSD­(T) reference data.^30^ On the other hand, the potentials are overestimated, leading to significant errors in the asymptotic region. The only exception is observed for LC-ωPBE DFA, where we note improved behavior due to its correct long-range description, i.e., 100% nonlocal HF exchange. This was also reported in refs 30 and 34. Similar observations apply to the correlated densities, which are obtained from fully self-consistent KS-DFT GKS calculations. All correlated densities are in phase with the reference CCSD­(T) data, although the quantitative agreement varies for all DFAs. The same behavior is visible for other atomic systems reported in SI. Slightly worse behavior is observed in molecular systems, with a more significant deviation in the core regions, consistent with other studies.^34,36,53^

**Figure 2:**
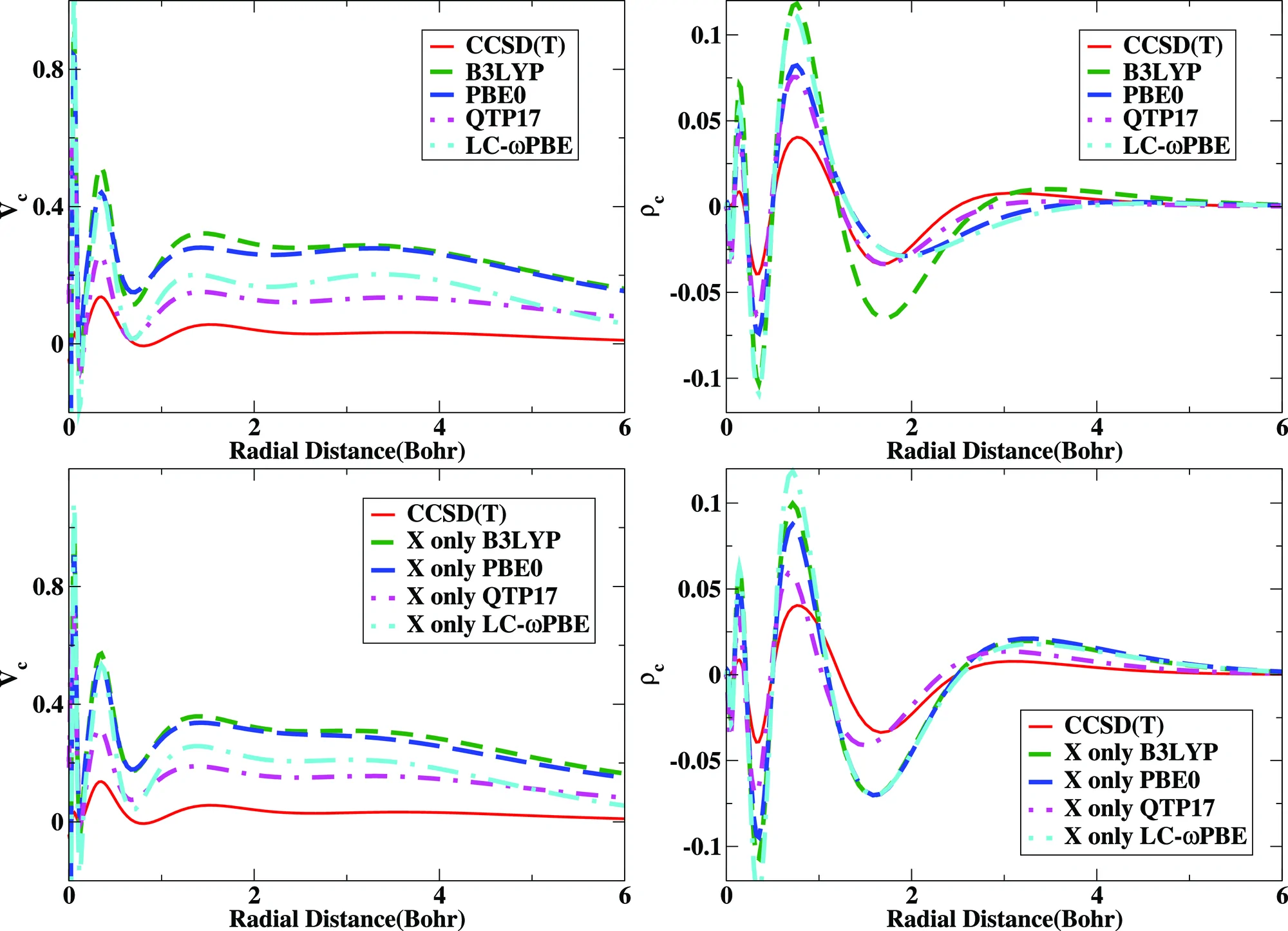
Correlation potentials (eq 9) and correlated densities (eq 8) of the Ar atom, calculated for several popular HYB DFAs with (top panel) and without (bottom panel) semilocal DFA correlation functional (*E*_*c*_^DFA^[ρ]) in eq 6.

In the bottom panel of Figure 2, we present the correlation potentials and correlated densities obtained from the self-consistent, exchange-only (X-only) HYB DFA calculations i.e., the semilocal DFA (*E*_*c*_^DFA^[ρ]) term is fully neglected in eq 6. Inspection of Figure 2 reveals that

(i) the correlation potentials and correlated densities obtained from the X-only case are qualitatively comparable to the reference data and to the full XC counterpart. This indicates that the *E*_*c*_^XDiff^[ρ] term in eq 7, which is mostly positive and formally depends on the X-only terms, carries a large portion of the correlation effects. It is evident that it plays a crucial role in restoring the correct shape and qualitative behavior of the correlation potential and correlated densities in the HYB DFAs
(ii) in principle, for the true exact density dependent exchange DFT functional, the *E*_*c*_^XDiff^[ρ] term as well as its functional derivative should be exactly zero. This is, for example, the case for the *ab initio* OEP DFT^54–56^ class of XC approximations, which has been discussed to some extent in the literature.^35,36^ Thus, the semilocal and HF exchange difference (*E*_*c*_^XDiff^[ρ]) can be used not only as a measure of the inaccuracy of the semilocal exchange energy approximation but also as an indicator of the accuracy of the exchange potential which, as we can observe here, evidently includes correlation effects too.

To verify that this trend persists for standard exchange-only DFA functionals, Figure 3 shows *v*_*c*_ and the DRDs for the Ar atom, obtained from self-consistent exchange-only calculations with the most commonly used exchange functionals, namely LDA, PBE, B88, TPSS, and R2SCAN, using eqs 8 and 9. In this case, correlation effects are substantially overestimated, which justifies scaling this contribution in eq 7 within the HYB class of functionals. Even so, the qualitative behavior remains very close to that of the reference data. These results further confirm that widely used standard DFT exchange functionals, in addition to their nominal exchange role, also encode significant amounts of correlation effects.

**Figure 3:**
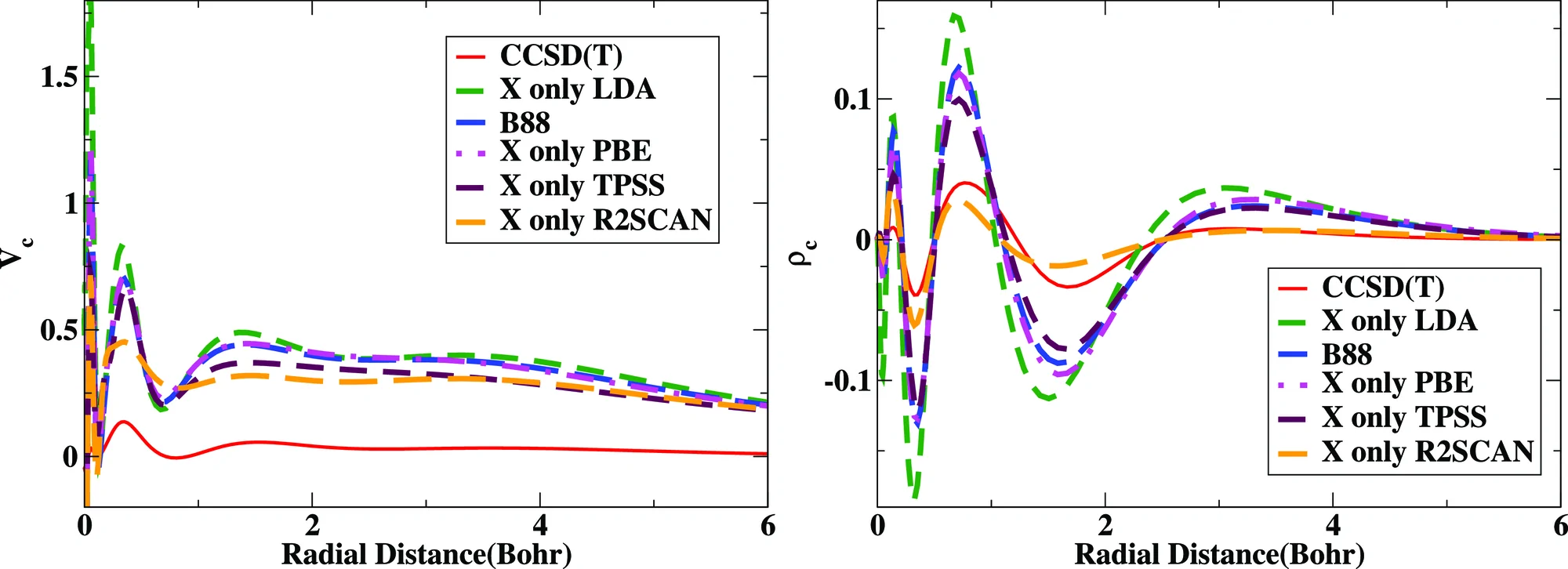
Correlation potentials (eq 9) and correlated densities (eq 8) of the Ar atom computed from standard semilocal X-only DFA functionals.

Furthermore, to enable a quantitative comparison of HYB DFAs, we analyze the effect of the semilocal *E*_*c*_^DFA^[ρ] term in eq 7 on self-consistent quantities. To this end, we employ the recently developed error metrics introduced in ref 30 to directly assess the quality of the XC and correlation KS potentials, electron densities, total energies, and ionization potentials (IPs) derived from the HOMO energy (see ref 30 for details). The resulting error values for the XC and exchange-only HYB DFAs are reported in Table 2.

**Table 2:** Average Errors for Different Physical Quantities for Full and Exchange-only Parts of Hybrid Functionals^a^

	XC						X-only					
	Δ*v*_*xc*_	Δ*v*_*c*_	Δ*ρ*	Δ*E*_*t*_	Δ*IP*	Δ*ρ* ^*ref*^ *v*_*xc*_	Δ*v*_*xc*_	Δ*v*_*c*_	Δ*ρ*	Δ*E*_*t*_	Δ*IP*	Δ*ρ* ^*ref*^ *v*_*xc*_
PBE0	0.667	9.973	0.002	0.001	0.259	0.092	0.671	10.060	0.004	0.008	0.32	0.106
B3LYP	0.713	10.710	0.004	0.002	0.276	0.103	0.72	10.773	0.004	0.008	0.346	0.111
QTP17	0.334	5.118	0.005	0.004	0.085	0.059	0.343	5.275	0.006	0.014	0.157	0.073
LC-ωPBE	0.118	1.863	0.004	0.001	0.057	0.102	0.130	2.063	0.005	0.006	0.118	0.117
HFLYP	0.088	1.266	0.003	0.001	0.099	0.029	0.092	1.0	0.003	0.007	0.061	0.017
UMK26	0.223	3.544	0.002	0.001	0.035	0.034	0.220	3.301	0.002	0.007	0.084	0.038
UMK26x							0.103	1.528	0.003	0.007	0.038	0.025

a The error metrics are elaborated in ref 30.

One can note that the average density (Δ*ρ*) and XC potential (Δ*v*_*xc*_) errors barely change between the XC and X-only cases. The same is visible for the average correlation potential (Δ*v*_*c*_) errors. This indicates a significant similarity in the correlation potentials between XC and X-only cases, especially in the asymptotic region.^30^ In the core and valence region (Δ*ρ* ^*ref*^ *v*_*xc*_ indicator), X-only *v*_*c*_ potentials give slightly larger errors, which is consistent with previous visual analysis. A similar behavior is also observed in the case of IP error (Δ*IP*). Here, the inclusion of the *E*_*c*_^DFA^[ρ] term improves in most cases (except HFLYP) the IPs of about ∼6–7% in MARE (∼0.03–0.04 E_*h*_ in MAE) computed with respect to IP-EOM-CCSD data in comparison to the X-only case (see Table S1 in SI file). This indicates that the semilocal *v*_*c*_^DFA^ contribution improves the full HYB correlation potentials in the core and valence regions, which impacts the HOMO energy quality. This is because the most semilocal correlation potentials yield a negative contribution in this region,^34,35^ which reduces the substantial positive overestimation of the correlation effects represented by the XDiff part.

The most significant deviations are visible on Δ*E*_*t*_ errors once the *E*_*c*_^DFA^ [ρ] term in the energy expression is neglected. However, this behavior is not surprising since we miss a dominant part of the correlation energy expression (see Table 1). Nonetheless, the above analysis clearly shows that the difference between the semilocal and exact-exchange potentials (*v*_*x*_^DFA^ – *v*_*x*_^HF^) behaves qualitatively similarly to the reference correlation potential. This property is crucial for the proper restoration of these effects in all HYB DFAs, which can partially explain the large success of this type of DFA.

At this point, we would like to emphasize that the Δ*v*_*c*_ correlation-potential error indicator and methodology introduced in ref 30 enable the assessment of how correlation effects are encoded in present-day semilocal DFA exchange-only KS potentials. To this end, we examined all semilocal exchange-only functionals available in the LIBXC library (version 7.0.0)^57^ and evaluated the extent to which they incorporate correlation effects by computing their correlation potentials, as defined in eq 9. In most cases, we find Δ*v*_*c*_ errors in the range of 12.3 to 14.4, values that are very similar to those of the worst-performing hybrid functionals reported in ref 30 (see the Δ*v*_*c*_ results in Table 1 of ref 30). For example, widely used exchange functionals such as LDA, B88,^58^ PBE,^59^ TPSS,^60^ and R2SCAN^61^ yield Δ*v*_*c*_ errors of 12.97, 12.95, 12.91, 12.79, and 12.77, respectively. This is further illustrated in Figure 3 (left), which shows the correlation potentials for the Ar atom obtained from self-consistent X-only DFA calculations using eq 9. Their qualitative behavior is very similar to that of the reference CCSD­(T) correlation potential, although their magnitudes are substantially overestimated. We may therefore conclude that all semilocal X-only functionals incorporate a significant portion of the true correlation effect. This observation is also consistent with earlier density-based analyses.^35,36,48,62^

Let us now turn our attention to eq 6 but with *n* = 1. In that case, HYB DFA functional is the simple sum of 100% nonlocal HF exchange functional and semilocal DFA correlation functional, *E*_*xc*_^HYB^[ρ] = *E*_*x*_^EXX^ + *E*_*c*_^DFA^[ρ]. One example of this type of hybrid DFA is the HFLYP^63,64^ where LYP correlation with 100% HF is solved self-consistently in the Generalized KS scheme. As shown in ref 30, this construction yields the lowest errors in the XC and correlation potentials among all HYB DFAs assessed in ref 30. However, a closer look at the LYP correlation potentials reported in Figure 4 reveals that it fails to describe the reference correlation potential features.^34,35^ The same applies to correlated densities reported in the right panel. One can note that HFLYP DRDs are out of phase with the reference data.^35,36^ Moreover, as illustrated in Figure 4, other widely used correlation functionals, such as PW91, PBE, and R2SCAN, exhibit similarly unphysical behavior. In particular, their correlation potentials are predominantly negative, which leads to noticeable errors in the correlated electron densities. These results highlight the fundamental incompatibility between semilocal correlation DFA expressions and an HF-like energy framework. Nevertheless, despite their inadequate description of correlation effects, these functionals remain important because they partially compensate for the overestimated correlation contributions arising from the *E*_*c*_^XDiff^[ρ] term in the HYB *n* ≠ 1 construction. This interplay gives rise to the well-known error-cancellation mechanism^34^ that underlies standard DFAs and hybrid functionals.

**Figure 4:**
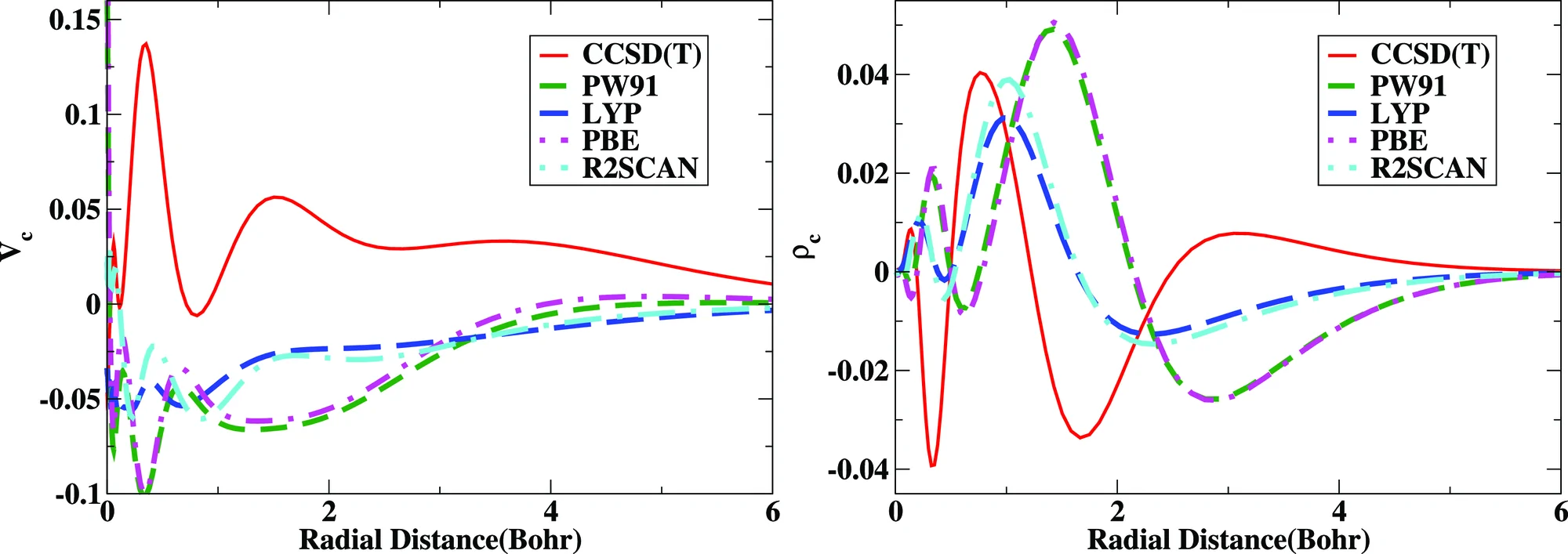
Correlation potentials (eq 9) and correlated densities (eq 8) computed from the self-consistent HF+ *E*_*c*_^DFA^[ρ] calculations for the Ar atom.

The above analysis clearly shows that HYB DFA XC functionals produce qualitatively correct correlation potentials, although their magnitudes are generally overestimated. We have also demonstrated that the XDiff term in eq 7 provides the key contribution determining both the shape of the correlation potential and the correlated density. This suggests that the semilocal exchange functional captures a large portion of the correlation effects, whereas semilocal correlation functionals represent them incorrectly.

Consequently, eq 6 for the case *n* ≠ 1 can be effectively employed as a constructive framework for achieving a qualitatively correct description of correlation effects in HYB DFAs. Furthermore, analysis of the XC potentials enables a direct interpretation of the (*n*–1) /*n* prefactor in terms of its role in representing correlation effects at the levels of energy, potential, and density. Therefore, eq 7 may be straightforwardly adopted as a practical recipe for constructing an EXX-compatible correlation energy functional within the HYB DFA framework.

This consideration brings us to the final part of the present study. Based on the conclusions drawn from the above analysis, we propose an interpretable and a physically motivated methodology for constructing new HYB DFA functionals as

10 $$E_{\mathrm{xc}}^{\mathrm{UMK}}\left[\rho \right]=E_{x}^{\mathrm{EXX}}+\gamma \left(E_{x}^{\mathrm{DFA}}\left[\rho \right]-E_{x}^{\mathrm{EXX}}\right)+E_{c}^{\mathrm{DFA}}\left[\rho \right]$$

where UMK stands for Uniwersytet Mikołaja Kopernika in Toruń (Nicolaus Copernicus University in Torun). In the construction defined by eq 10, the parameter γ = (*n* – 1)/*n* scales the difference between the semilocal and the exact-exchange HF parts. This term effectively captures the dominant part of the correlation effects incorporated in HYB DFAs through exchange mixing, providing a transparent physical interpretation of the hybrid construction. As a proof of concept, we implement this methodology using the B88 and LYP functionals for the semilocal exchange and correlation components, respectively. Nonetheless, the proposed framework is general, and other semilocal exchange and correlation functionals can be employed and tested within eq 10. In that case, the optimal value of γ may change and should be reoptimized.

The crucial step in our construction is the optimal choice of the γ parameter. Once again, we rely on the measures introduced in ref 30. Unfortunately, for global hybrid DFAs, the Δ*v*_*XC*_ and Δ*v*_*c*_ error indicators cannot be used directly, because they receive relatively large contributions from the incorrect asymptotic decay of the XC potential in the density tail. As an alternative, we consider HOMO ionization-potential energies, which have been shown to directly reflect the quality of the XC and correlation potentials^30,34,47,66^ (see, e.g., Figure 6 in ref 30). This optimization strategy can also be justified from the perspective of correlated orbital theory (COT).^67^ Therefore, the γ parameter in eq 10 was determined by a one-dimensional line-search procedure in the interval 0 ≤ γ ≤ 1. For each trial value of γ, fully self-consistent calculations were performed for 16 systems (listed in Table 3) of the CCSD­(T) benchmark set introduced in ref 30, computing their average Δ*IP* error. The minimum of this error (Δ*IP* = 0.035) curve was obtained for γ = 0.25, which defines the UMK26 functional. This is presented in Figure S17 in the SI file.

As demonstrated in ref 30 (see Figure 5), a larger fraction of HF exchange leads to a substantial reduction of the Δ*v*_*XC*_ error, which is consistent with our expectations. Consequently, this reduces the overestimation of correlation potentials in the core, valence, and asymptotic tail.

**Figure 5:**
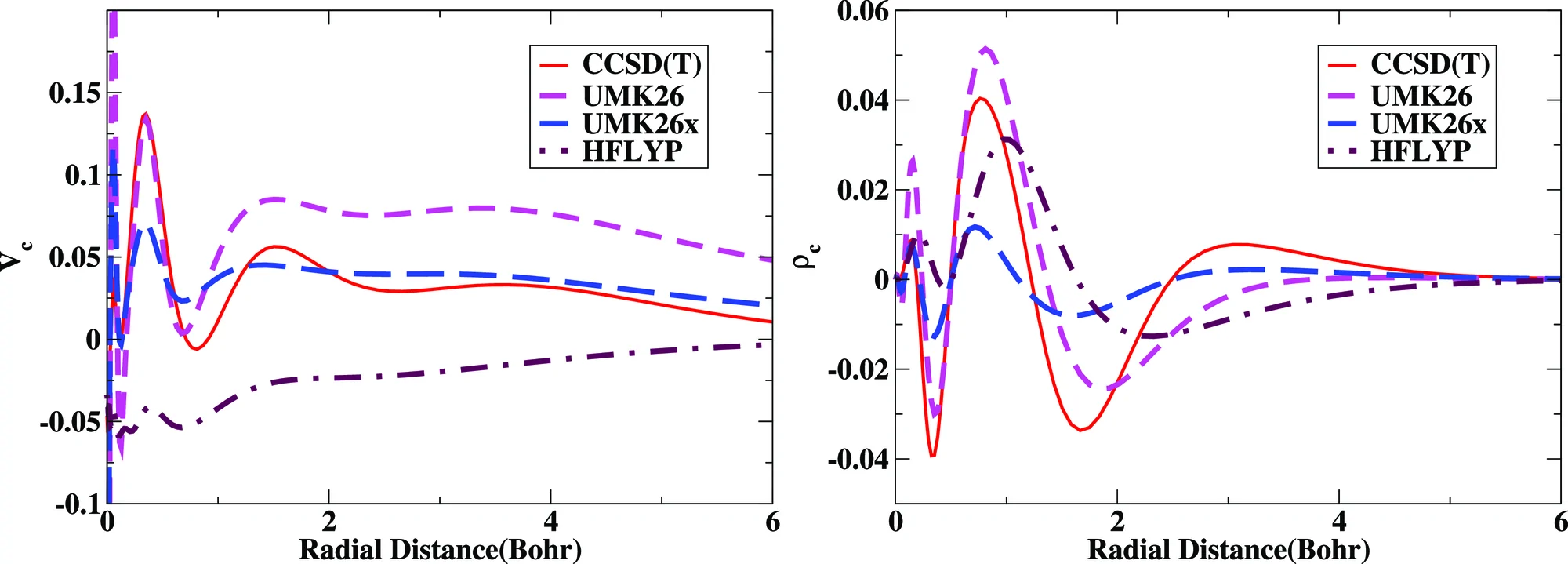
Correlation potentials and correlated densities calculated for the Ar atom using HFLYP, UMK26, and UMK26x, with respect to the reference CCSD­(T) plots.

We note also that while the precise value of γ is functional-dependent, we expect that the optimal scaling factor for many semilocal exchange functionals will remain close to the value obtained for the B88/LYP realization due to the similar overestimation of correlation effects carried by the functional itself (see Figure 3). As a test, we have performed the same minimalization analysis for a PBE-based UMK hybrid construction and obtained the minimum Δ*IP* at γ = 0.26, which is almost identical to the value found for UMK26; see also Figure S18 in the SI.

As shown in the first part of this paper, the majority of the correlation effects is carried by the XDiff term eq 6 of the HYB DFA functional. Thus, to evaluate the behavior at the level of pure exchange, we used eq 11 to define an additional variant of UMK26, denoted as UMK26x, in which the LYP correlation term is fully neglected, and the difference of the semilocal and HF exchange terms entirely represents the correlation effects i.e.,

11 $$E_{\mathrm{xc}}^{\mathrm{UMK}26x}\left[\rho \right]=E_{x}^{\mathrm{EXX}}+\gamma \left(E_{x}^{B88}\left[\rho \right]-E_{x}^{\mathrm{EXX}}\right)$$

using the same γ-optimization procedure, we found the optimal γ for the UMK26x variant to be 0.1, indicating the dominant role of HF exchange.

To assess the performance of the UMK26 and UMK26x HYB DFA functionals, we first analyze the corresponding correlation potentials and correlated densities computed for a representative set of atomic and molecular systems (see also the SI). As shown in Figure 5, the correlation potentials obtained with the UMK26 and UMK26x functionals are qualitatively consistent with the CCSD­(T) reference potential. Importantly, we observe a substantial reduction in the overestimation of the correlation potentials characteristic of standard HYB DFAs (see Figures 2 and 3 for the exchange-only case). This clearly demonstrates that the proposed methodology performs as intended. A similar trend is observed for the correlated densities obtained from fully self-consistent UMK26 and UMK26x calculations. The resulting densities are in phase with the CCSD­(T) reference data and reproduce both their spatial profiles and magnitudes. This agreement is particularly pronounced for the UMK26 functional. Comparable improvements are found for other systems, including Be, Ne, CO, and H_2_O (see Figures S13–S16). Nevertheless, noticeable deviations remain in the asymptotic region of the correlation potentials. These discrepancies arise from the different asymptotic decay behaviors of the semilocal and exact-exchange components of the potential.^30^ Addressing this limitation will be the subject of future work.

Next, we apply the UMK26 and UMK26x functionals to directly calculate IPs from orbital energies in fully self-consistent hybrid DFT calculations. To this end, we consider two widely used and well-established benchmark sets: the GW100 test set^68^ and the IP benchmark set developed by Bartlett’s group.^66^ We note that the GW100 set contains 12 of the 16 systems used in the γ line-search optimization. However, GW100 itself was not used during the optimization, and the reported GW100 statistics are evaluated over the complete benchmark set, which contains a much broader and chemically more diverse collection of systems. Therefore, do not expect this partial overlap to introduce a significant bias in the GW100 assessment.

For the GW100 benchmark set, we compute the mean absolute deviation (MAD) of the HOMO energies (HOMO IPs) obtained with the GKS and G_0_W_0_ approaches for our new functionals, as well as for several other popular global and RS hybrids. The errors are evaluated relative to the CCSD­(T)/def2-TZVPP reference data^68^ and are reported in Table 3. Notably, the global hybrid UMK26 yields a very low MAD of 0.38 eV, substantially outperforming other global hybrids and most RS counterparts, while remaining comparable to the best range-separated QTP variants.^66^ A similar trend is observed for UMK26x, which also performs well, with a relatively small MAD of 0.48 eV. These results indicate that reducing the overestimation of the potential has a strong positive effect on the quality of the HOMO orbital energies, even though the XC potential still does not recover the correct asymptotic – 1/*r* behavior in the tail region. Consistent with this, G_0_W_0_ calculations based on both global and RS hybrids also demonstrate excellent performance for UMK26 and UMK26x. In particular, UMK26 achieves a MAD of 0.18 eV at the G_0_W_0_ level, again matching the accuracy of the QTP family of functionals, which represent the leading RS hybrid functionals for reliable IP prediction.

**Table 3:** Mean Absolute Deviation (MAD) (in eV) on GW100 Reference Data Set Computed Using HOMO Energies Obtained with GKS and G_0_W_0_ Approaches^a^

	GKS	G_0_W_0_
UMK26x	0.48	0.27
UMK26	0.38	0.18
PBE0	2.63	0.33
B3LYP	2.90	0.38
QTP17	0.71	0.15
HFLYP	1.30	0.25
LC-ωPBE	0.66	0.29
LC-ωPBEH	0.86	0.29
CAM-B3LYP	1.33	0.30
QTP00	0.33	0.31
QTP01	0.34	0.16
QTP02	0.31	0.19

a The errors are computed with respect to CCSD­(T)/def2-TZVPP reference data.

As an additional assessment, we consider the benchmark set from ref 66, which, in addition to HOMO ionization potentials, also enables the evaluation of valence IPs. The results obtained with the same global and range-separated hybrid functionals used for the GW100 set are summarized in Table 4. For the HOMO IPs, UMK26 and UMK26x follow the same trend observed previously, outperforming conventional global hybrids and some range-separated hybrids, and achieving an accuracy comparable to that of the QTP family of functionals. For the valence IP benchmark, UMK26’s performance deteriorates somewhat; however, the results remain qualitatively comparable to those of QTP00. UMK26x, in turn, shows slightly worse performance than UMK26, although it still outperforms LC-ωPBEH and CAM-B3LYP. Given that UMK26 functionals are global hybrids that effectively compete with RS variants in describing IPs, these findings are highly encouraging.

**Table 4:** Mean Absolute Deviation (MAD) (in eV) Calculated the Benchmark Set from Ref 66 for Several Global and Range-separated Hybrid Functionals with Respect to IP-EOM-CCSD/cc-pVTZ Data^a^

	MAD HOMO	MAD val.
UMK26x	0.53	1.00
UMK26	0.42	0.75
PBE0	2.97	3.10
B3LYP	3.23	3.44
QTP17	0.79	0.47
HFLYP	1.51	2.58
LC-ωPBE	0.31	0.39
LC-ωPBEH	1.09	1.17
CAM-B3LYP	1.53	1.58
QTP00	0.32	0.65
QTP01	0.29	0.30
QTP02	0.24	0.35
HF	0.74	1.68

a At the bottom, we report the HF data for comparison.

One of the fundamental issues in KS-DFT is the integer (derivative) discontinuity, which arises when electrons are removed from or added to a chemical species. The exact XC functional is expected to yield a total energy that varies linearly with the electron number between systems with integer electron occupations, i.e., it should produce a straight line connecting the energies of the systems with *N*_0_, *N*_0_ – 1, and *N*_0_ + 1 electrons.

Figure 6a illustrates this behavior for the fluorine atom (number of electrons, *N* = 9) and its cationic (*N* = 8) and anionic (*N* = 10) forms, showing how the total energies of the fractional-electron systems deviate from the ideal piecewise linear interpolation defined by the corresponding integer-electron energies for a few representative DFAs. Both PBE0 and B3LYP present convex curvature in the electron removal and electron addition regions. In these cases, the deviation is more pronounced under ionization.

**Figure 6:**
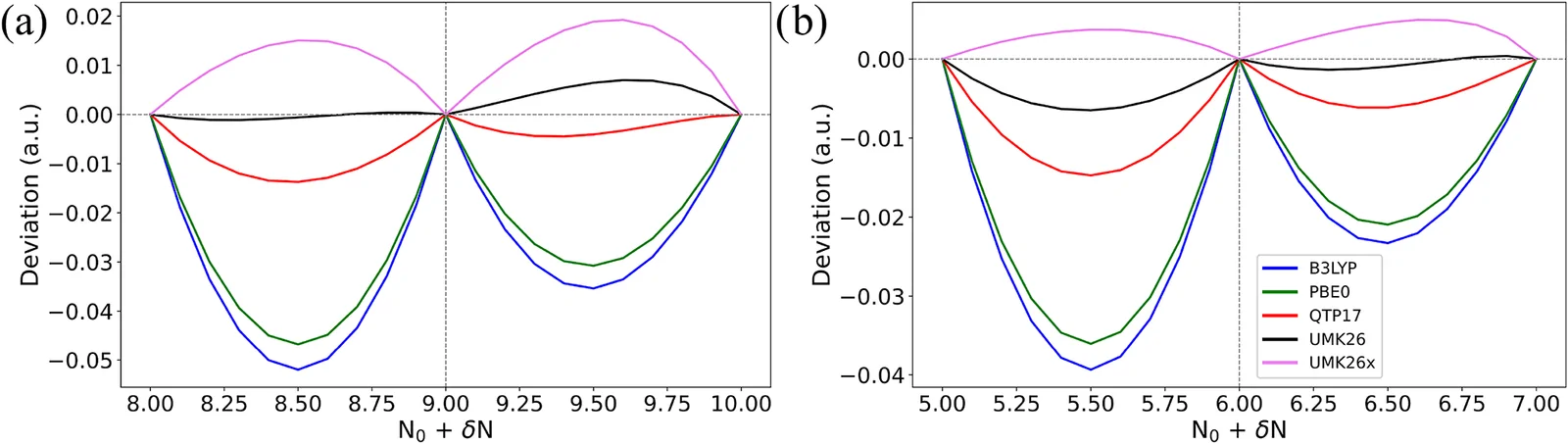
Deviation of hybrid XC functionals from the piecewise linear interpolation regarding the fractional electron occupancies of (a) fluorine atom and (b) carbon atom.

A similar trend is observed for the QTP17 functional; however, its deviation from linearity is significantly smaller. QTP17 also performs notably well in describing electron attachment. It is noteworthy that the hybrid parameters of QTP17 were optimized to reproduce all five experimental ionization potentials of the water molecule,^51^ following the correlated orbital theory’s IP condition of Bartlett,^67^ which states that the negative of each occupied orbital eigenvalue should correspond to its respective IPs. In contrast, as mentioned previously, UMK26 parameters were optimized using only the first ionization potentials (HOMO IPs) of a series of several chemical species.

In this context, UMK26x exhibits concave curvature in both the electron addition and electron removal regions, as indicated by the violet curve in Figure 6. Here, the error is slightly larger in the ionization description. Finally, UMK26 (black line) shows excellent performance in the electron removal region, producing an almost perfectly linear interpolation. Although its description of electron attachment is not strictly linear, the deviation is substantially lower than for all other hybrid functionals (except QTP17). This behavior indicates nearly negligible localization/delocalization errors during ionization and minimal errors upon electron attachment.

In the case of the carbon atom (Figure 6b), a similar trend is observed overall. However, UMK26 performs better in the electron attachment region, while UMK26x is as good as or slightly better in the electron removal region.

We remark that the integer discontinuity error is known to lead to a poor performance of KS-DFT for charge-transfer (CT) excitations.^69,70^ For example, at a large separation of the donor–acceptor (DA) species, the excitation energy of a CT state can be approximated as $\omega_{\mathrm{CT}}={\mathrm{IP}}_{D}-{\mathrm{EA}}_{A}-\frac{1}{R_{\mathrm{DA}}}$ .^71^ Therefore, UMK26 is an excellent candidate for future photophysical studies of systems containing excited states exhibiting CT character.

To verify the previous statement, Table 5 shows how UMK26 and the other functionals perform for long-range charge transfer, i.e., when 1/*r*_*DA*_ is very small. In this situation, the charge transfer can be approximated by the simple expression ω_CT_ = *IP*_D_ – *EA*_A_. For this analysis, we used the test set from Verma et al.,^72^ in which the NH_3_···HNO_2_ dimer is evaluated at nine intermolecular distances ranging from 3.68 to 25.76 Å. With a MAD of 0.49 eV, UMK26 is by far the best-performing global hybrid functional. The second-best result is obtained with QTP17, which has a MAD of 1.68 eV (1.19 eV higher!). Moreover, UMK26 outperforms most RS Hybrid functionals, except for QTP00 and QTP02, which yield MADs of 0.37 and 0.46 eV, respectively.

**Table 5:** Vertical Excitation Energies (eV) for Long-Range Charge-Transfer Excited States of the NH_3_···HNO_2_ Dimer Obtained in TDDFT Calculations with the jul-cc-pVTZ Basis Set and Deviations with Respect to Reference Results from Verma et al.^72^

1/*R*_*DA*_	ref	PBE0	B3LYP	UMK26	QTP17	LC-ωPBE	LRC-ωPBEH	CAM-B3LYP	QTP00	QTP01	QTP02
3.6772	7.10	3.95	3.56	7.46	6.48	6.74	5.15	5.24	7.17	6.49	6.87
6.1133	8.22	3.90	3.44	8.04	6.89	7.69	5.98	5.70	7.97	7.41	7.79
8.5632	8.75	3.97	3.49	8.42	7.18	8.41	6.50	6.02	8.44	7.94	8.32
11.016	9.13	4.03	3.53	8.65	7.37	8.53	6.82	6.21	8.73	8.26	8.64
13.471	9.35	4.07	3.56	8.80	7.49	8.74	7.04	6.34	8.92	8.47	8.85
15.927	9.51	4.10	3.58	8.92	7.58	8.90	7.19	6.44	9.06	8.62	9.00
18.387	9.62	4.13	3.60	9.00	7.65	9.01	7.30	6.51	9.16	8.73	9.11
23.303	9.77	4.17	3.63	9.11	7.75	9.16	7.46	6.61	9.30	8.89	9.27
25.763	9.83	4.18	3.64	9.16	7.78	9.22	7.51	6.65	9.35	8.94	9.33
MAD		4.98	5.47	0.49	1.68	0.56	2.26	2.84	0.37	0.84	0.46
MAX		5.65	6.19	0.67	2.05	0.61	2.32	3.18	0.48	0.89	0.51
MSD		–4.98	–5.47	–0.41	–1.68	–0.56	–2.26	–2.84	–0.35	–0.84	–0.46

Next, Figure 7 presents a CT analysis for excitations across different dimers at a fixed distance. In this case, the description of CT states is no longer given simply by the difference between IPs and EAs. Under these conditions, UMK26 stands out as the best-performing functional overall, with a MAD of 0.74 eV. The only other functionals achieving MADs below 1 eV belong to the QTP family (MADs ranging from 0.81 to 0.96 eV),except for QTP01 (MAD = 1.10 eV).

**Figure 7:**
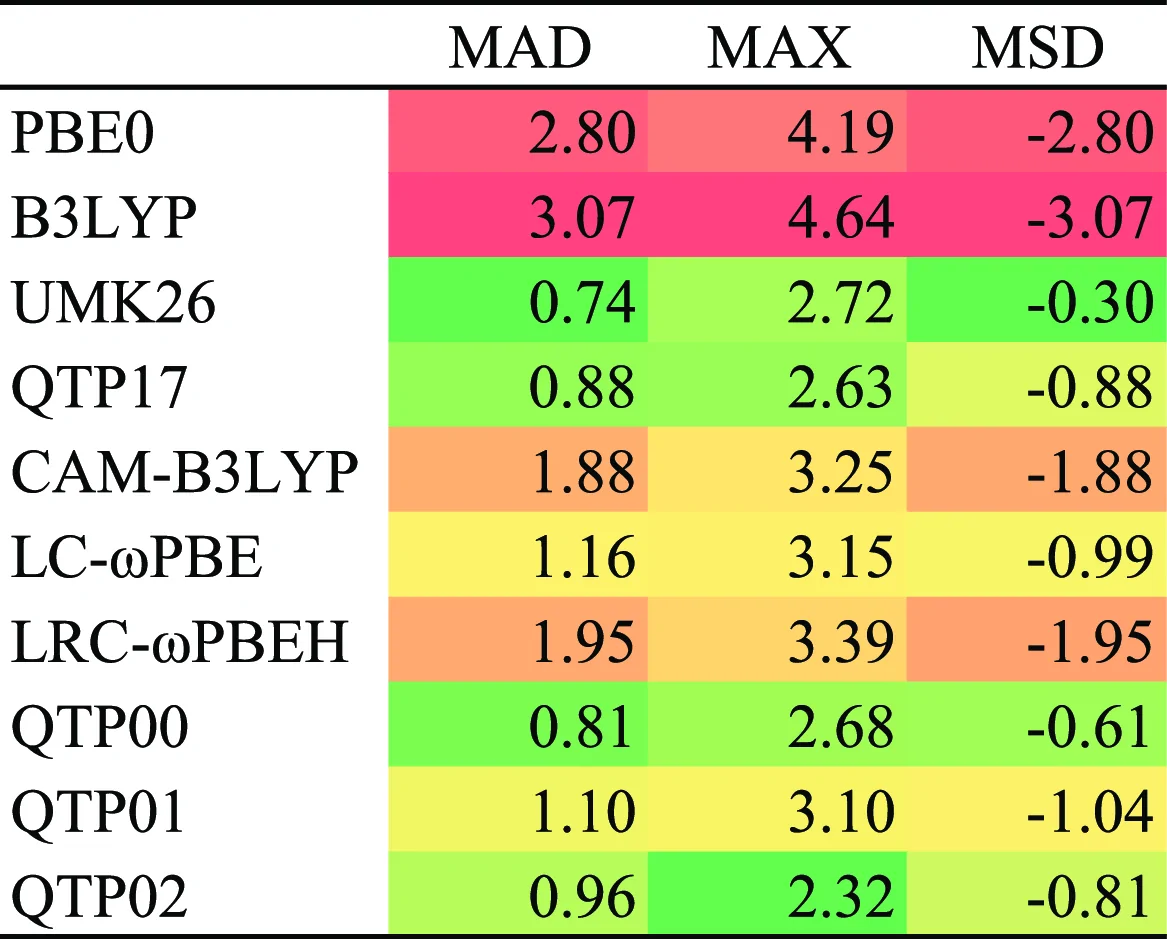
Heat map showing the mean absolute deviation (MAD) and mean signed deviation (MSD), in eV, of the calculated charge-transfer excited states with respect to EOM-CCSDT-3 reference data from Kozma et al.^73^ The cc-pVDZ basis set was used.

For completeness, a final test on CT states is presented in Figure 8. This set comprises 19 organic molecules exhibiting intramolecular charge transfer. In this case, CAM-B3LYP performs best among the selected functionals, with a MAD of 0.23 eV. Mester and Kállay^74^ also reported this favorable performance, although their study employed the TDA approach. Among the global hybrids, the best performer is QTP17 (MAD = 0.33 eV), while UMK26 shows similar performance, with a MAD of 0.38 eV. Interestingly, the “exchange-only” variant, UMK26x, yields a considerably good result, with a MAD of 0.67 eV. Despite containing no correlation (functional) contribution, its performance is comparable to, or even slightly better than, that of widely used XC functionals such as B3LYP (MAD = 0.74 eV) and PBE0 (MAD = 0.64 eV), for example.

**Figure 8:**
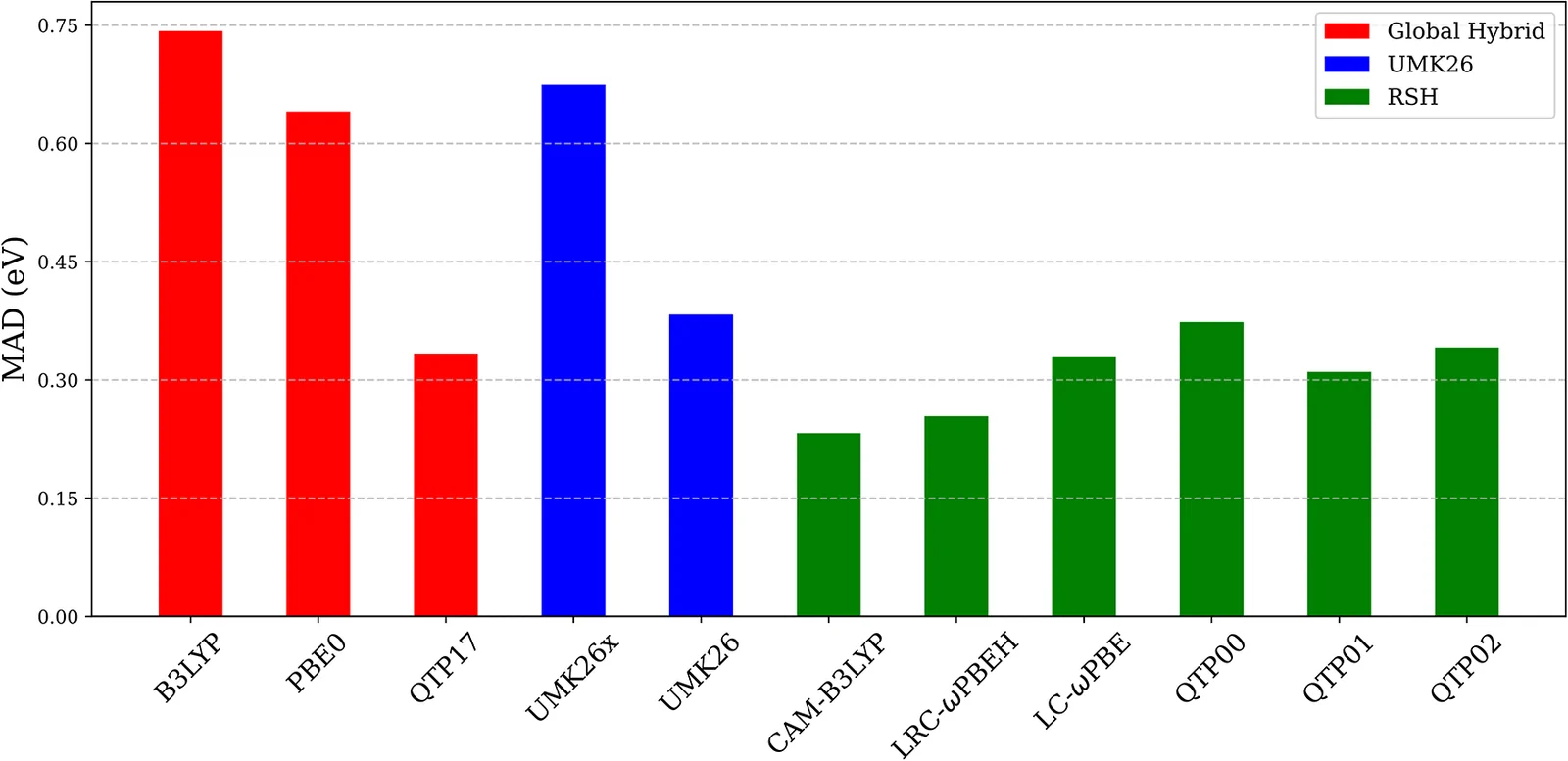
Mean absolute deviations (MADs) of intramolecular charge-transfer excitations w.r.t theoretical best estimates (TBE) from Loos et al.^75^ The basis set was cc-pVTZ.

As a final observation, all functionals underestimate intermolecular CT excitations relative to the reference values (see the MSD values in Table 5 and Figure 7). For intramolecular CT excitation, however, although the overall trend remains (underestimation), the “IP corrected functionals”, i.e., QTP family and UMK26 variants, exhibit the opposite behavior and instead overestimate these results (see Table S5 in SI).

As a final part of this paper, we assess the performance of UMK26 on energy and energy-related data sets.^76,77^ To this end, we employ the recently proposed SLIM20 data set^78^ developed to mimic the statistics of the large GMTKN55 data set^76^ and enables a faster assessment of exchange-correlation functionals.

The results collected in Figure 9 show that UMK26 exhibits a highly nonuniform but physically meaningful performance pattern on the SLIM20 benchmark. In terms of the overall WTMAD-2, UMK26 gives 12.46 kcal/mol, which is better than HFLYP (14.13) and close to B3LYP (12.84), but still worse than PBE0(10.31) and more balanced RSHs, such as QTP17 (9.77), QTP02 (9.23), and LC-ωPBE (10.06). Thus, UMK26 is clearly not the best general-purpose functional within the present set as judged solely by the global SLIM20 aggregate. The functional is clearly weaker across several thermochemical and bond-dissociation subsets, including ALKBDE10 (19.90), YBDE18 (6.40), G21EA (9.13), G21IP (6.09), W4–11 (35.32), and especially MB16–43 (51.84). It is also not among the best methods for subsets such as WATER27 or PA26. However, one can note that this simple global-hybrid construction performs exceptionally well in situations dominated by self-interaction and delocalization errors. This is most evident from the SIE4×4 subset in Figure 9, where UMK26 yields by far the lowest MAD, 0.65 kcal/mol, outperforming PBE0 (9.83), B3LYP (11.47), HFLYP (4.00), QTP17 (2.67), QTP02 (4.47), and LC-ωPBE (5.83). Such behavior is particularly noteworthy because UMK26 does not use the additional flexibility of range separation and nevertheless surpasses the other tested hybrid forms for these strongly self-interaction-sensitive systems. In addition to SIE4×4, UMK26 is also the best performer for PX13 (2.51) and ISO34 (0.37), and remains very competitive for FH51 (1.01), IL16 (1.19), RSE43 (0.62), and TAUT15 (0.22). These trends consistently indicate that the UMK26 DFA effectively suppresses spurious delocalization and improves the description of difficult energetic balances involving localized charge distributions, consistent with the conclusions drawn from Figure 6.

**Figure 9:**
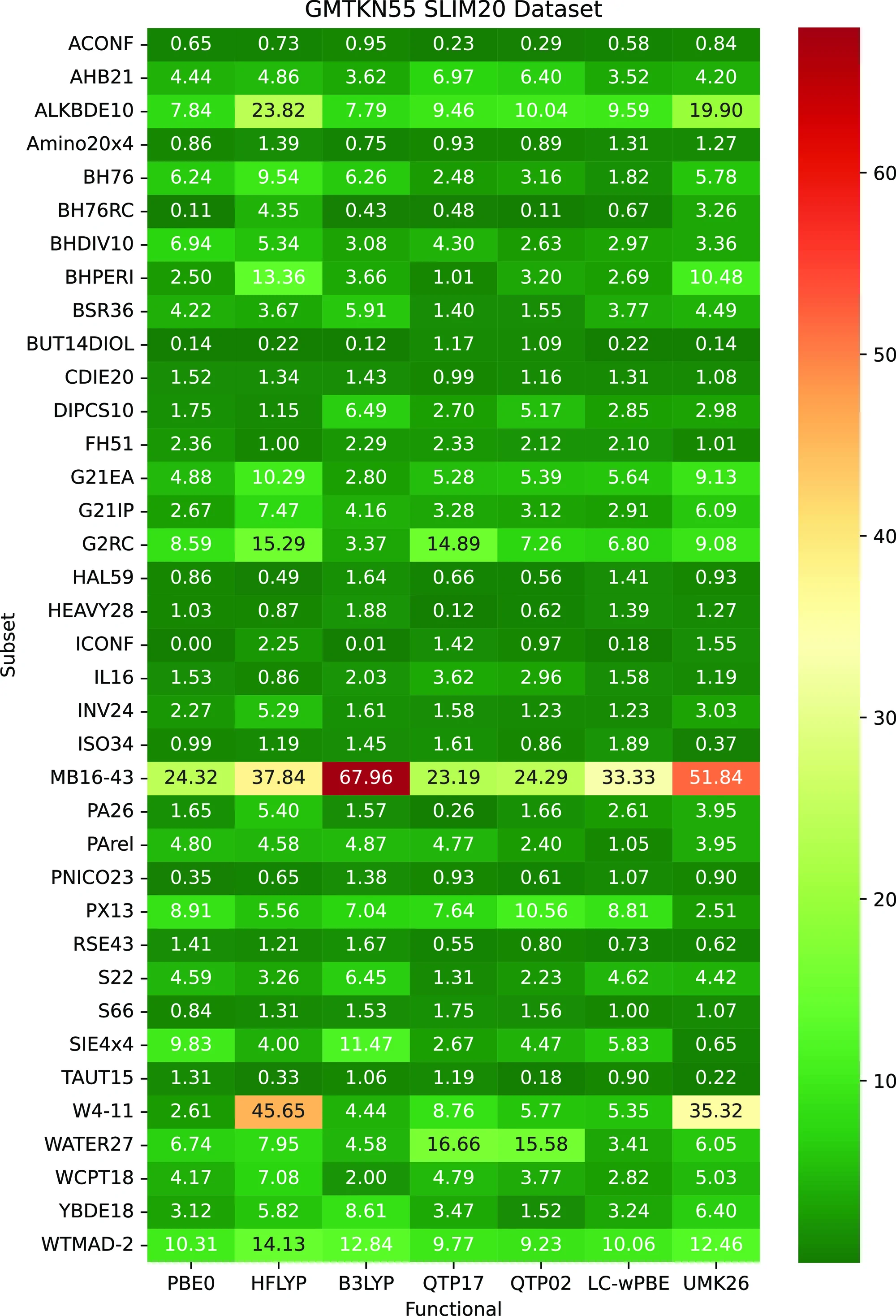
Mean absolute deviation for GMTKN55 SLIM20 data set. def2-TZVP basis set is employed here, and the values are reported in kcal/mol. WTMAD-2 is computed with the original GMTKN55 formula with MAD from SLIM20 subsets.

The physical origin of this behavior is directly illustrated by the dissociation curves in Figure 10. For H_2_^+^ and He_2_^+^, approximate functionals affected by self-interaction error tend to show an unphysical residual attraction at large interatomic separation, reflecting an incorrect stabilization of fractionally delocalized charge. In both systems, UMK26 reduces this spurious long-range binding more effectively than the other DFAs considered. The effect is especially clear for He_2_^+^, where UMK26 approaches the correct dissociation limit substantially better than PBE0, QTP17, QTP02, and LC-ωPBE. A similar tendency is also observed for ArKr^+^, where UMK26 avoids the severe overbinding seen with PBE0 and follows the reference curve more closely in the asymptotic region. These results provide direct molecular-level confirmation of the SIE4×4 benchmark trend, i.e., the excellent performance of UMK26 for self-interaction-sensitive subsets is not accidental, but reflects a genuine improvement in the dissociation behavior of charged systems.

**Figure 10:**
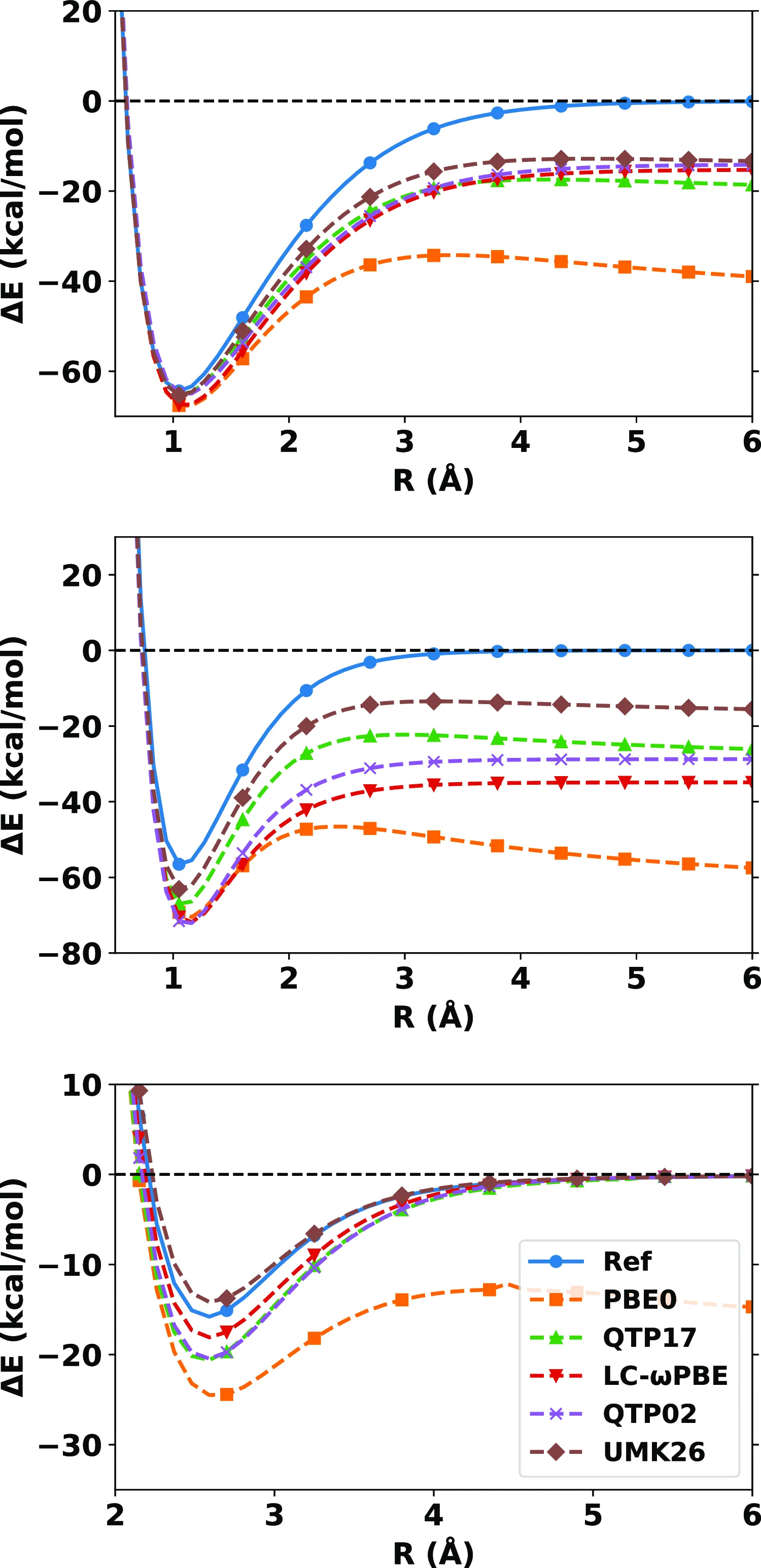
Dissociation Curve for *H*_2_^+^(Top), *He*_2_^+^(Middle) and ArKr^+^(Bottom). The Def2-QZVP basis set is employed together with HF and CCSD­(T) reference (Ref) for *H*_2_^+^, and *He*_2_^+^(Middle), ArKr^+^, respectively.

At the same time, the significance of UMK26 extends beyond self-interaction tests alone. As shown in Figure 11, UMK26 lies in a favorable region of the Δ*E*_*t*_-Δ*ρ* diagram,^30^ indicating a good compromise between density accuracy and total-energy accuracy. This is an important observation because, although UMK26 does not perform uniformly well across all SLIM20 subsets, its position in Figure 11 suggests that its total-energy description is quite good and, in fact, better balanced than for several other considered here DFAs. This finding is further supported by the AE18 subset, extracted from the MGCDB84 data set, which contains the absolute total atomic energies of the atoms from hydrogen through argon. For this subset, UMK26 again yields the lowest MAD, 8.21 kcal/mol, improving upon HFLYP (9.01) and clearly outperforming B3LYP (17.84), PBE0 (41.98), QTP02 (57.45), and especially QTP17 (183.84). Since AE18 directly probes the quality of absolute total energies, its favorable result for UMK26 is fully consistent with the Δ*E*_*t*_ analysis in Figure 11. Taken together, these two observations suggest that UMK26 benefits from a favorable energetic balance and, consequently, from a useful degree of error cancellation at the energy level. Thus, even though it is not the best functional for many chemically diverse subsets in SLIM20, the underlying total energies are described rather well, which can partly compensate for more localized deficiencies in specific reaction or bonding categories.

**Figure 11:**
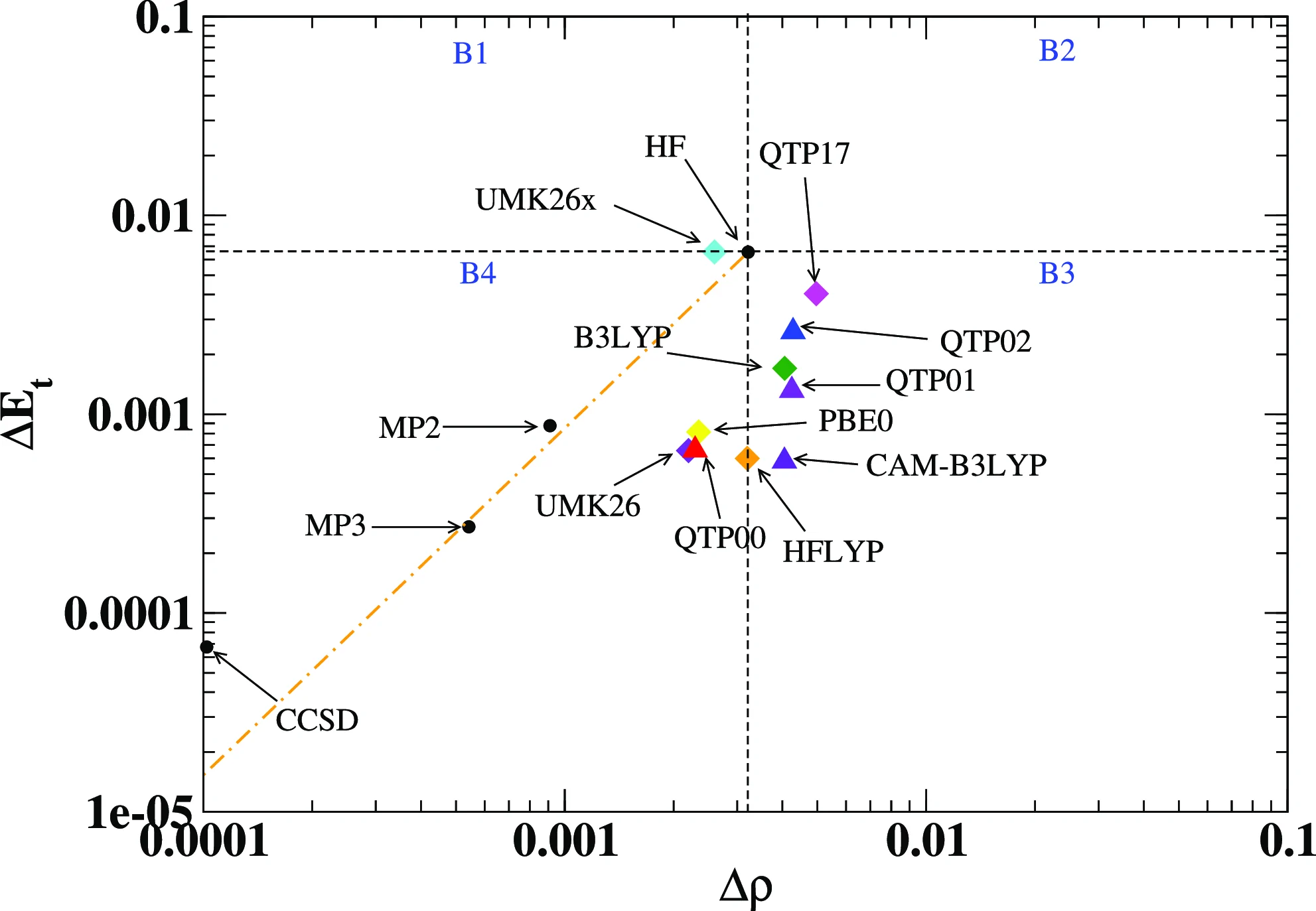
A comparison of Δ*ρ* vs Δ*E*_*t*_ for UMK26 and UMK26x with other well-known global and range-separated hybrid functionals studied in ref 30.

Therefore, UMK26 cannot be regarded as a uniformly superior replacement for the better-balanced RSH DFAs in broad benchmark applications. Nevertheless, the present results show that UMK26 combines a simple global-hybrid form with excellent performance in SIE-dominated cases and a robust description of total energies, making it a promising, physically well-motivated construction.

## 4 Conclusions

In this work, we analyzed how correlation effects are represented in hybrid density functional approximations from the perspectives of the KS XC potential, correlated densities, and energy-related observables. The present results show that, within the hybrid framework, the dominant qualitative features of correlation are not generated primarily by the semilocal correlation functional alone, but rather by the difference between the semilocal exchange and exact-exchange contributions. This term accounts for a substantial portion of the correlation effect and is essential for restoring the physically correct structure of the correlation component of the full XC potential and the corresponding correlated density. In this sense, the semilocal part of hybrid functionals plays a much deeper role than that suggested by a purely energetic decomposition, since it directly controls how correlation is represented in the local *v*_*xc*_ potential.

Our analysis further shows that semilocal correlation functionals applied on top of full exact-exchange are not compatible with a physically correct description of correlation effects in the potential. Their role in standard hybrid constructions is largely compensatory, i.e., they reduce the systematic overestimation of correlation encoded by the exchange-difference contribution, especially in the core and valence regions. This provides a transparent interpretation of conventional hybrid mixing and explains why hybrid DFAs can produce qualitatively meaningful correlation potentials even when neither constituent part is satisfactory on its own. On this basis, we propose an interpretable construction principle for hybrid functionals in which the key ingredient is the controlled scaling of the semilocal/exact-exchange difference term.

As a proof of concept, this methodology was used to construct the simple global hybrid UMK26, together with its exchange-only counterpart UMK26x. Despite their uncomplicated global-hybrid form, both functionals substantially reduce the overestimation of correlation effects observed in standard hybrids and yield correlation potentials and correlated densities that closely follow the high-level reference behavior. Most importantly, UMK26 delivers very accurate IPs from orbital energies and shows excellent performance for CT excitations, placing it on a comparable footing with several recently proposed RSHs. This is a particularly encouraging result because it demonstrates that a physically motivated global hybrid, designed through analysis of the XC potential rather than empirical energy fitting alone, can recover much of the performance often associated with more flexible range-separated forms.

At the same time, the present study clarifies both the physical interpretability and the limitations of the proposed construction. The introduction of parameter γ provides a physically interpretable strategy for controlling the semilocal/exact-exchange difference and, consequently, the representation of correlation in the XC potential and correlated density. However, its optimized value is generally system-, property-dependent. This dependence reflects the approximate DFA ingredients used in the construction and residual error-cancellation effects, which are not yet controlled systematically. Thus, although the construction principle in eq 10 is physically motivated, a single value of γ cannot be expected to provide universally reliable results across different systems and properties. Further limitations arise from the asymptotic behavior associated with the DFA ingredients and the exact-exchange fraction. In particular, the 75% exact-exchange coefficient in UMK26 produces an asymptotic −0.75/*r* tail rather than the exact −1/*r* behavior. These limitations motivate future work on range-separated extensions, additional DFA ingredients, basis-set effects, systematic control of error cancellation, and potential- and density-guided optimization of γ.

We believe that this perspective opens a useful route for the rational development of future hybrid approximations, including improved global hybrids, range-separated hybrids, and double-hybrid forms built on the same physical principles. In the latter case, the present analysis should apply directly to the hybrid part of conventional double hybrids, while the effect of the additional perturbative correlation contribution on the density and local potential^79,80^ remains an important subject for future work.

The present framework also provides a natural target for future ML-assisted hybrid-functional design. Unlike recent adaptive hybrid approaches,^25^ which predict system-dependent exact-exchange fractions mainly from energetic and electronic-property targets, the present analysis points to training objectives based directly on XC potentials, correlation potentials, correlated densities, and IP-related indicators.

## 5 Computational Details

PySCF (version 2.9)^81,82^ and KS-Pies^83^ package is employed for the generation of the Wu-Yang XC potential and electron densities at the DFT level. The reference potential and densities at the CCSD­(T) level were computed using the PSI4 package^84^ and the n2v^85^ package. An uncontracted aug-cc-pVTZ^86^ basis set is used to expand both orbitals and potentials to generate the XC potential and density on a 3D DFT grid. The trust-exact optimization procedure with the Fermi-Amaldi guide is employed in the Wu-Yang inverse procedure. The guide potential is scaled with respect to the long-range Hartree–Fock coefficient in the case of hybrid density functionals, and a regularization value of 1 × 10^–5^ is used to avoid artificial oscillations in the core of the XC potential. The PySCF package is used to benchmark against the GMTKN55 SLIM20 ^78^ data set with the def2-TZVP basis set,^87^ and to generate dissociation curves as depicted in Figure 10 with the def2-QZVP basis.^88^ The PSI4 package with cc-pVTZ basis is employed to benchmark the HOMO and valence IPs against the IP-EOM-CCSD reference^66^ as depicted in Table 4. For the GW100 benchmark,^68^ the def2-TZVPP basis set was employed, which requires effective core potentials (ECPs) for heavy elements. In this case the GKS and *G*_0_W_0_ data have been obtained with MOLGW code.^89^ The fractional occupations were also performed with PSI4 using the aug-cc-pVTZ basis set.^86,90,91^ Charge-transfer calculations were performed using a combination of PSI4 (Tables 5 and Figure 7) and the Orca package^100^ (Figure 8). For these charge-transfer calculations, the jul-cc-pVTZ,^92^ cc-pVDZ,^86,90^ and cc-pVTZ basis sets were employed, respectively.

## Supplementary Material



## Data Availability

The data underlying this study are available in the published article and the Supporting Information. Data have been generated using the software described in the Computational Details section.

## Notes

Additional information, such as plots for Ne, Be, CO, and H_2_O (PDF)
