# Supplementary material for: Primary Role of Semi-Local and Exact-Exchange Difference in the Description of Correlation Effects in Hybrid Functionals
Source: J Chem Theory Comput. 2026 Sep 21;22(19):10036–51. doi: 10.1021/acs.jctc.6c00843 (PMC13671180; doi:10.1021/acs.jctc.6c00843)
Supplement: Supplementary file 1 [file ct6c00843_si_001.pdf]

Supplementary Information for "Primary Role of Semi-Local and Exact-Exchange Difference in the Description of Correlation Effects in Hybrid Functionals"

Vignesh Balaji Kumar,[†] Szymon Śmiga,[*,†,‡] Rodrigo Araújo Mendes,[¶] Bogumiła Jezierska,[†] Rodney Bartlett,[¶] and Ireneusz Grabowski[*,†,‡]

†*Institute of Physics, Faculty of Physics, Astronomy, and Informatics, Nicolaus Copernicus University in Toruń, ul. Grudziądzka 5, 87-100 Toruń, Poland*

‡*Institute of Advanced Studies, Nicolaus Copernicus University in Toruń, ul. Wileńska 4, 87-100 Toruń, Poland*

¶*Quantum Theory Project, University of Florida, Gainesville, Florida 32611, United States*

E-mail: szsmiga@fizyka.umk.pl; ig@fizyka.umk.pl

I) Hybrid and Exchange only Hybrid Functionals

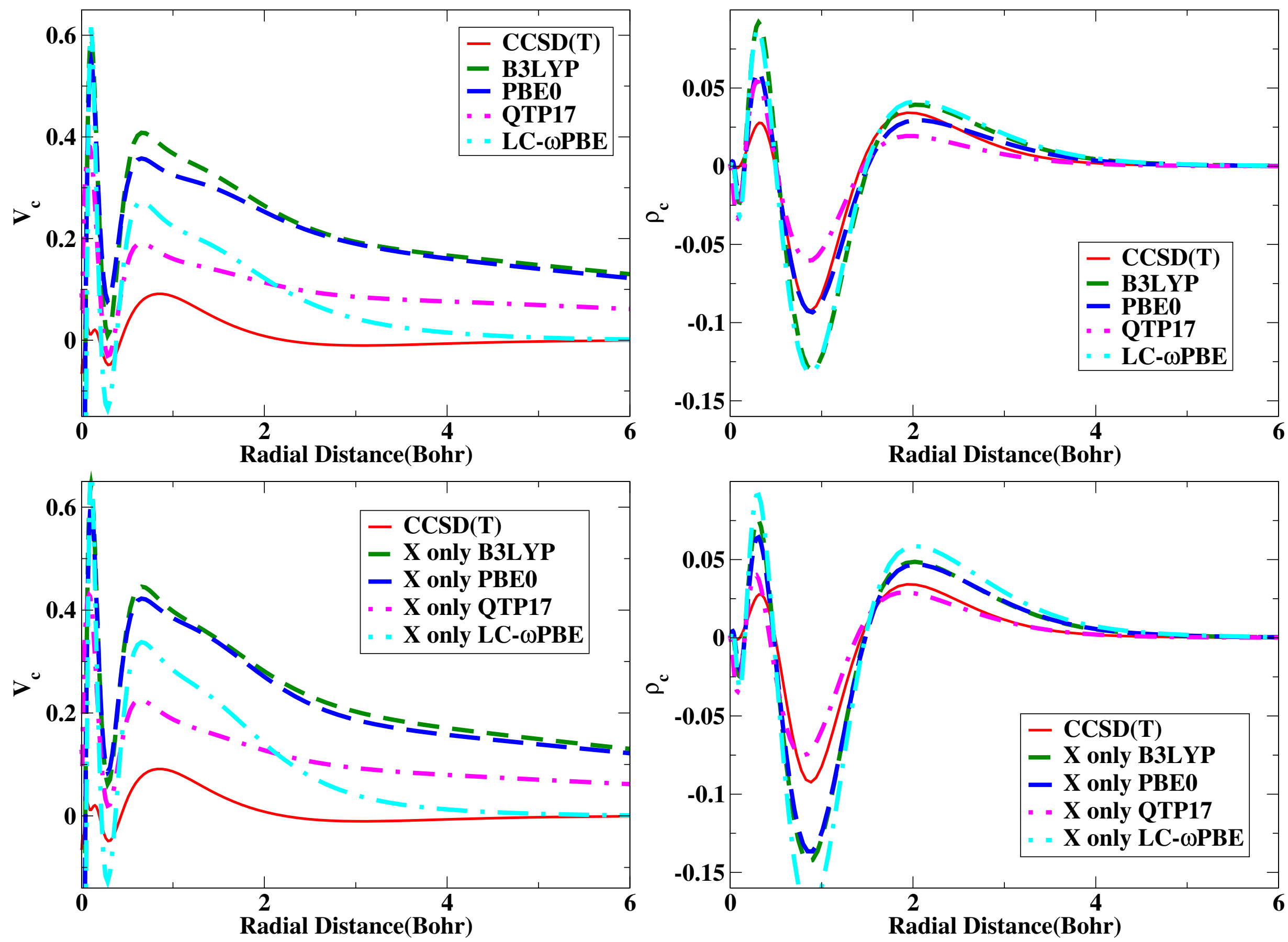

Figure S1: The correlation potentials (Eq. (9)) and correlated densities (Eq. (8)) of the Ne atom, calculated for several popular HYB DFAs with (top panel) and without (bottom panel) semi-local DFA correlation functional ($E_c^{\mathrm{DFA}}[\rho]$) in Eq. (6).

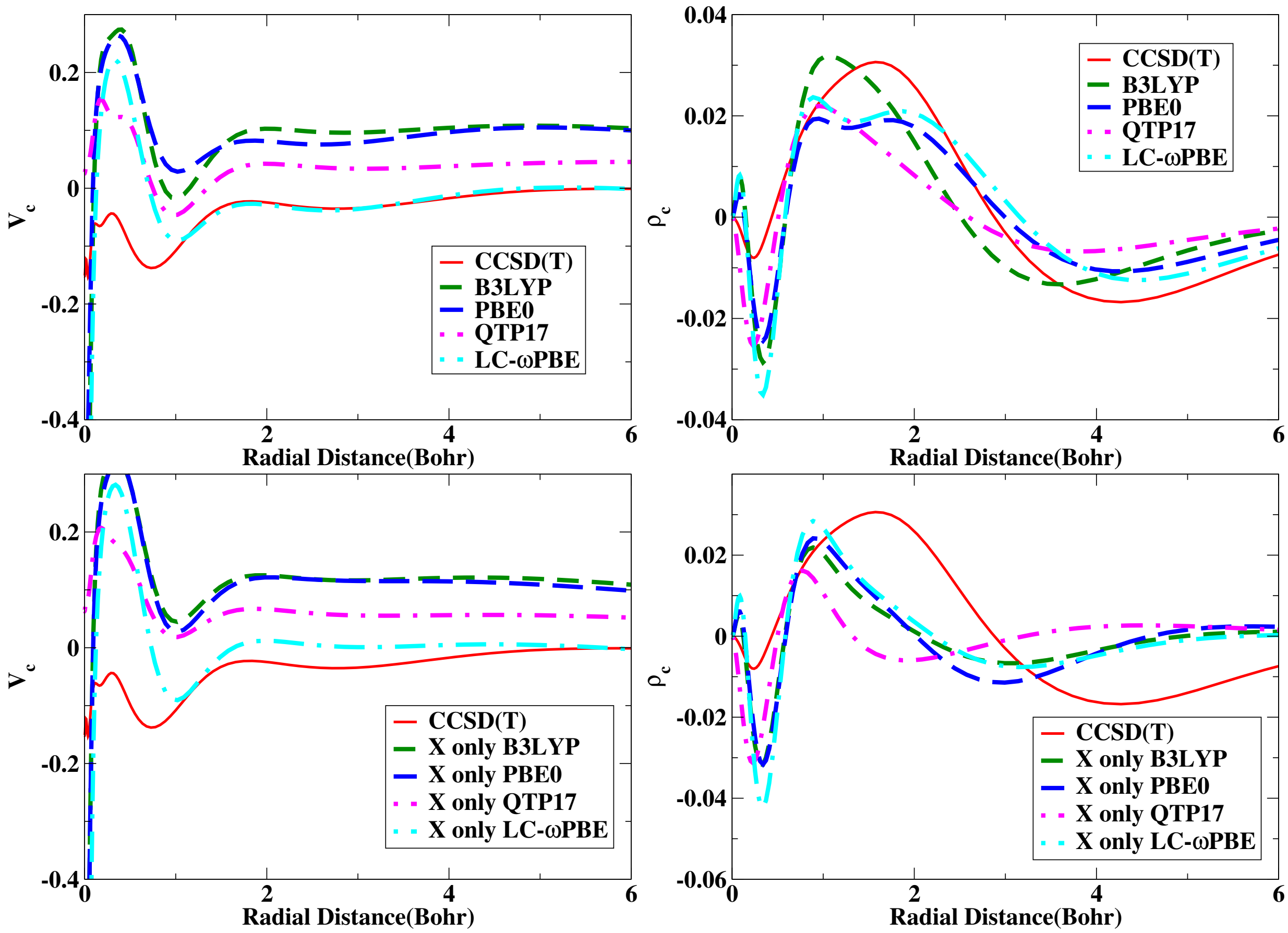

Figure S2: The correlation potentials (Eq. (9)) and correlated densities (Eq. (8)) of the Be atom, calculated for several popular HYB DFAs with (top panel) and without (bottom panel) semi-local DFA correlation functional ($E_c^{\mathrm{DFA}}[\rho]$) in Eq. (6).

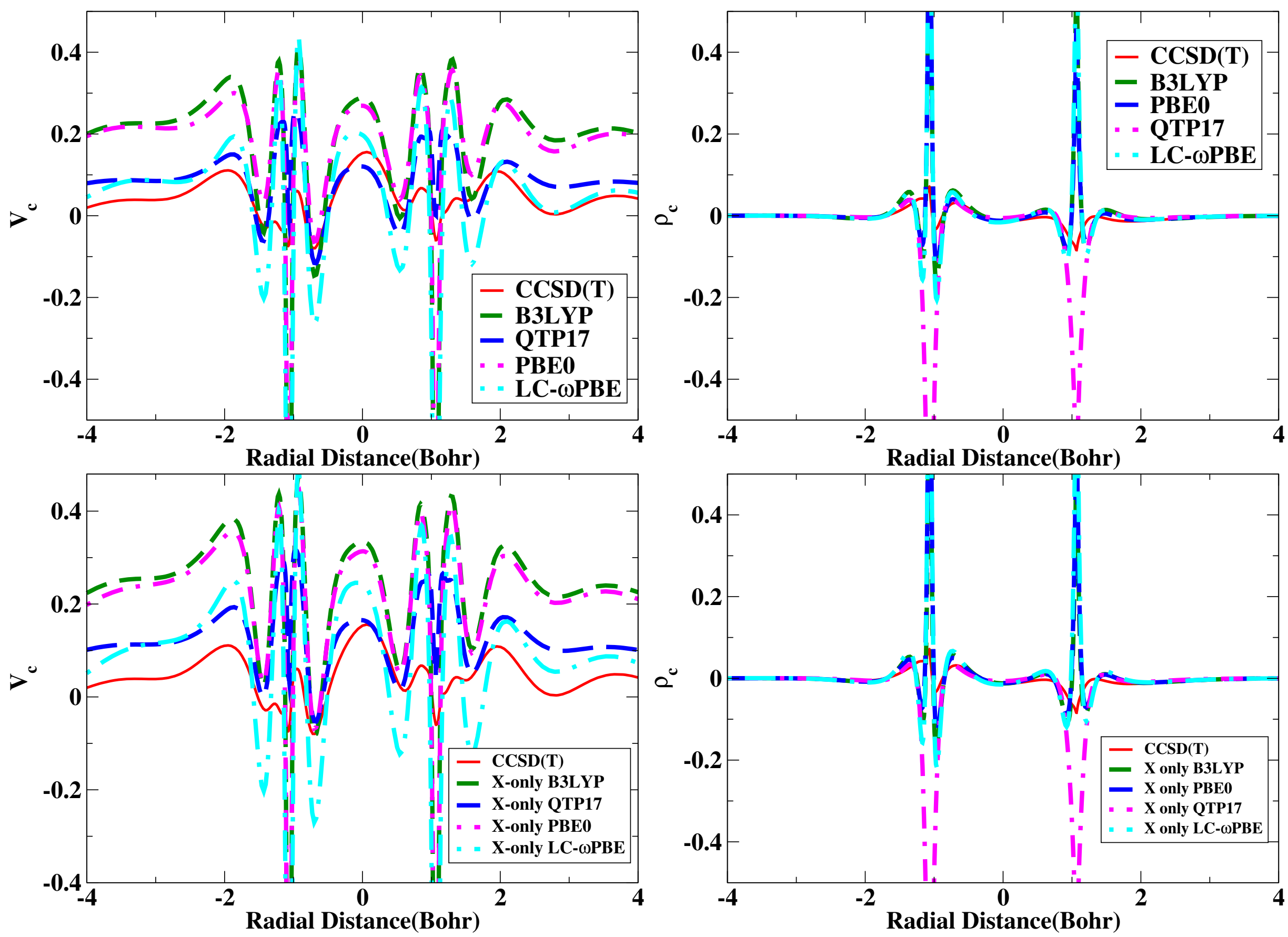

Figure S3: The correlation potentials (Eq. (9)) and correlated densities (Eq. (8)) of the CO molecule, calculated for several popular HYB DFAs with (top panel) and without (bottom panel) semi-local DFA correlation functional ($E_c^{\mathrm{DFA}}[\rho]$) in Eq. (6).

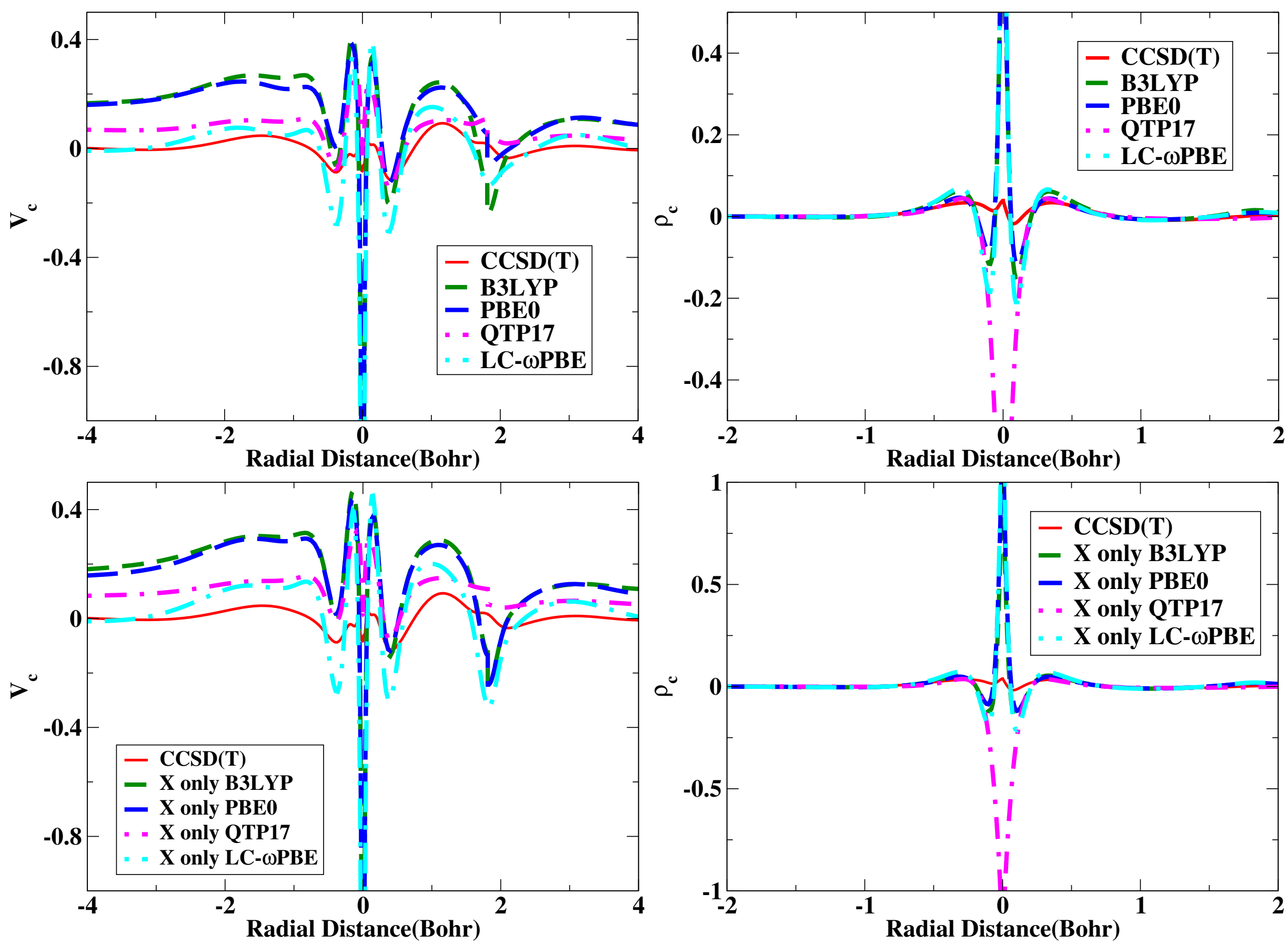

Figure S4: The correlation potentials (Eq. (9)) and correlated densities (Eq. (8)) of the H_2O molecule, calculated for several popular HYB DFAs with (top panel) and without (bottom panel) semi-local DFA correlation functional ($E_c^{\mathrm{DFA}}[\rho]$) in Eq. (6).

II) Exchange only Local/Semilocal Functionals

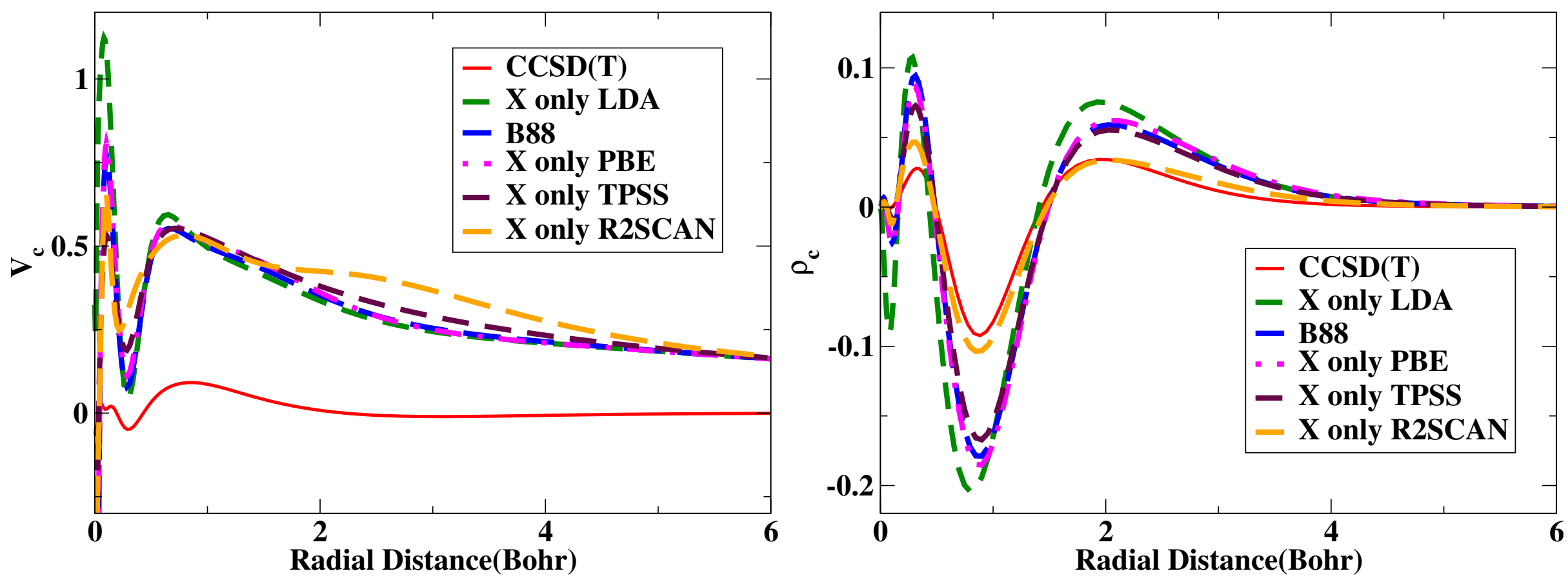

Figure S5: The correlation potentials (Eq. (9)) and correlated densities (Eq. (8)) of the Ne atom computed from standard semi-local X-only DFA functionals.

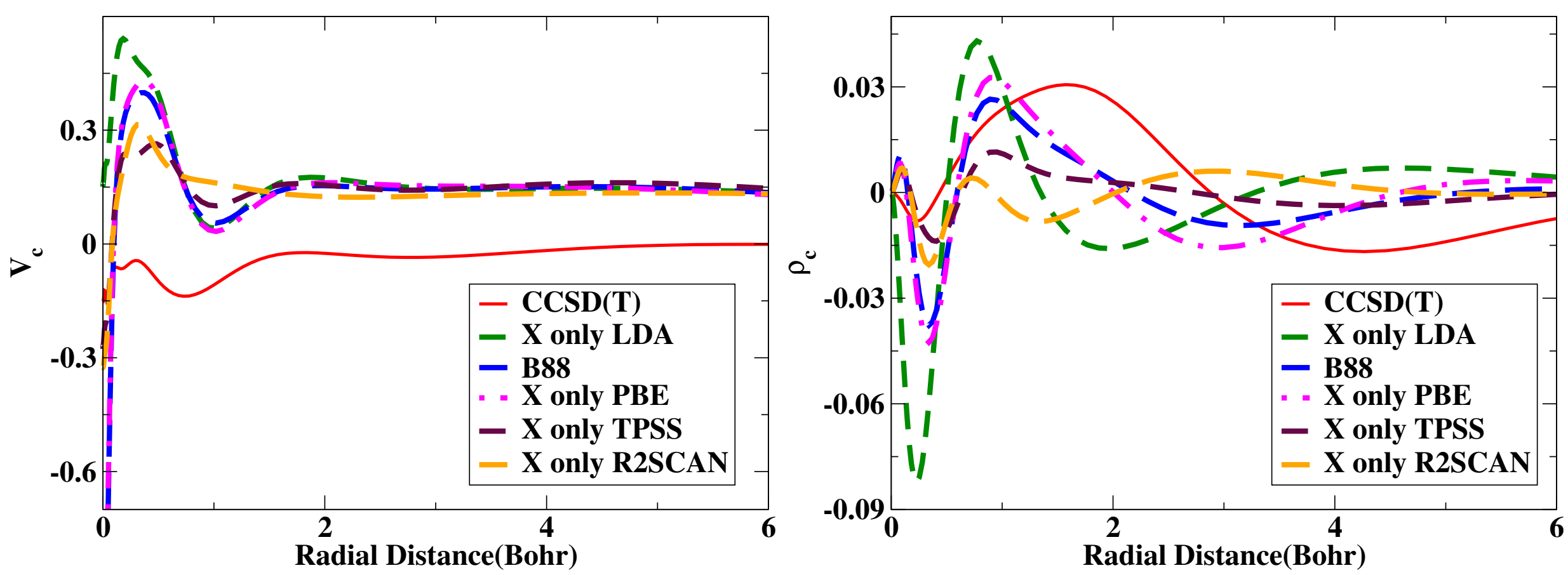

Figure S6: The correlation potentials (Eq. (9)) and correlated densities (Eq. (8)) of the Be atom computed from standard semi-local X-only DFA functionals.

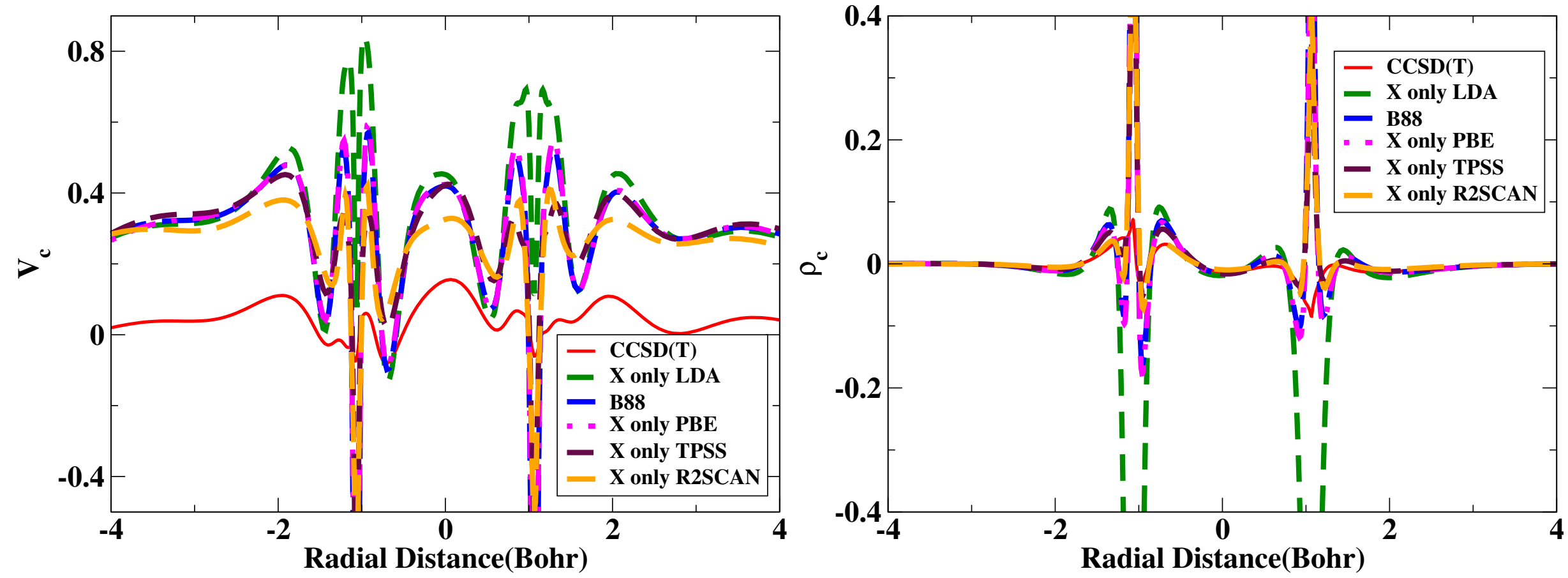

Figure S7: The correlation potentials (Eq. (9)) and correlated densities (Eq. (8)) of the CO molecule computed from standard semi-local X-only DFA functionals.

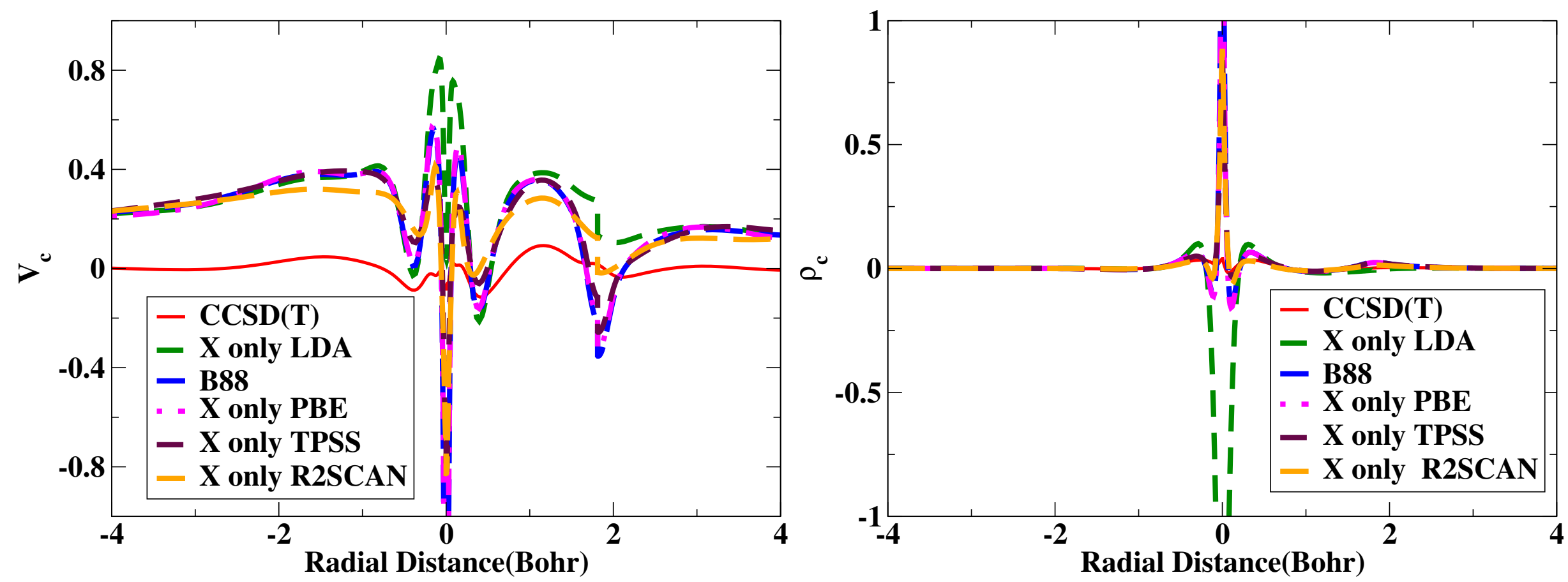

Figure S8: The correlation potentials (Eq. (9)) and correlated densities (Eq. (8)) of the H_2O molecule computed from standard semi-local X-only DFA functionals.

III) Semilocal correlation with 100% HF

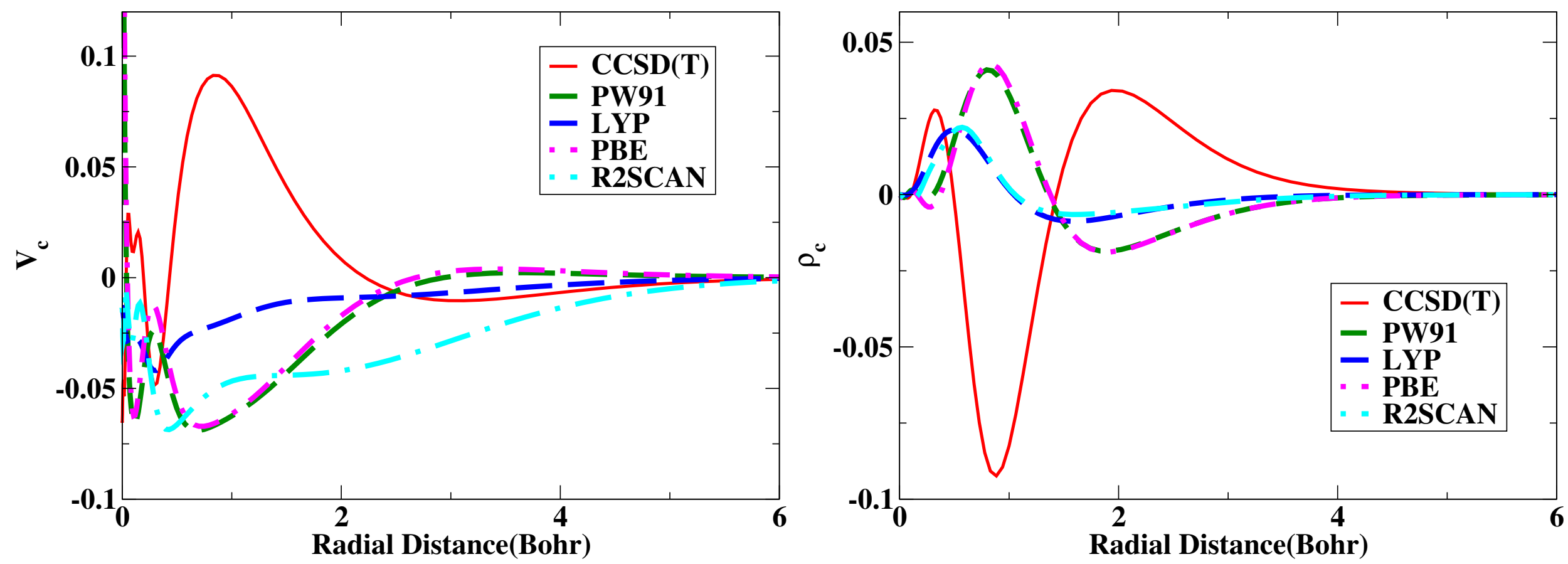

Figure S9: The correlation potentials (Eq. (9)) and correlated densities (Eq. (8)) computed from the self-consistent 100% HF+ $E_c^{\mathrm{DFA}}[\rho]$ calculations for Ne atom

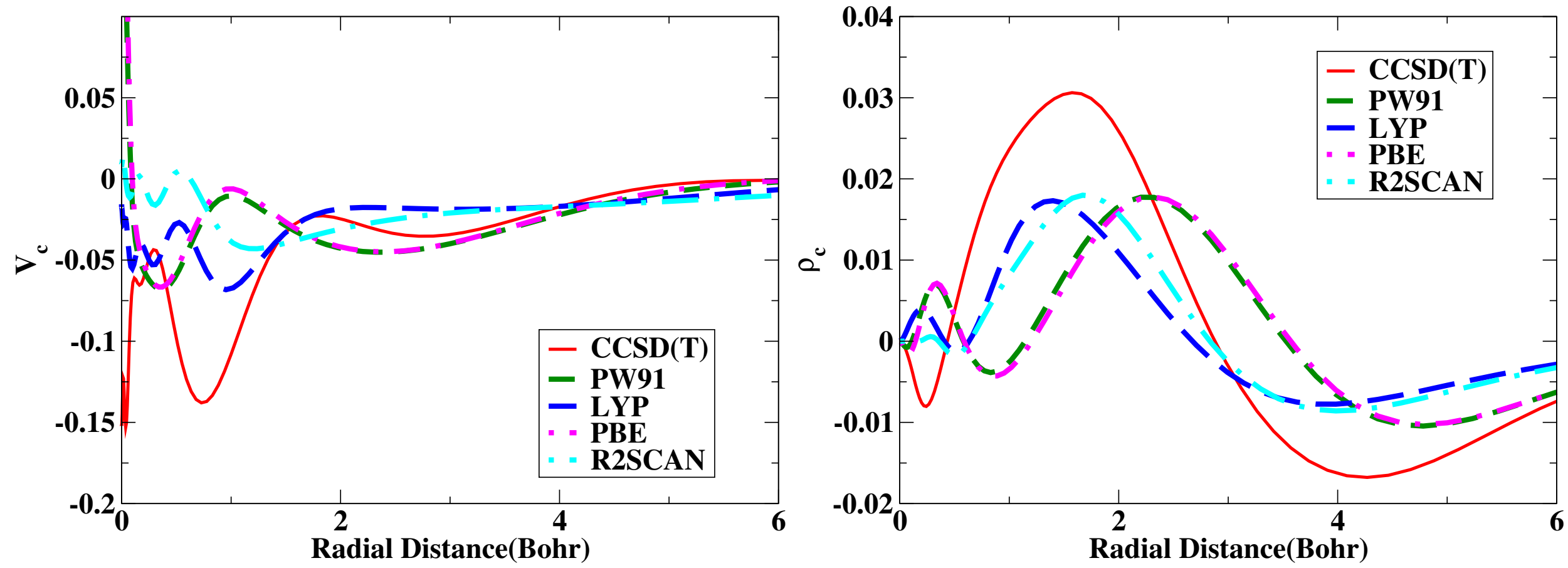

Figure S10: The correlation potentials (Eq. (9)) and correlated densities (Eq. (8)) computed from the self-consistent 100% HF+ $E_c^{\mathrm{DFA}}[\rho]$ calculations for Be atom

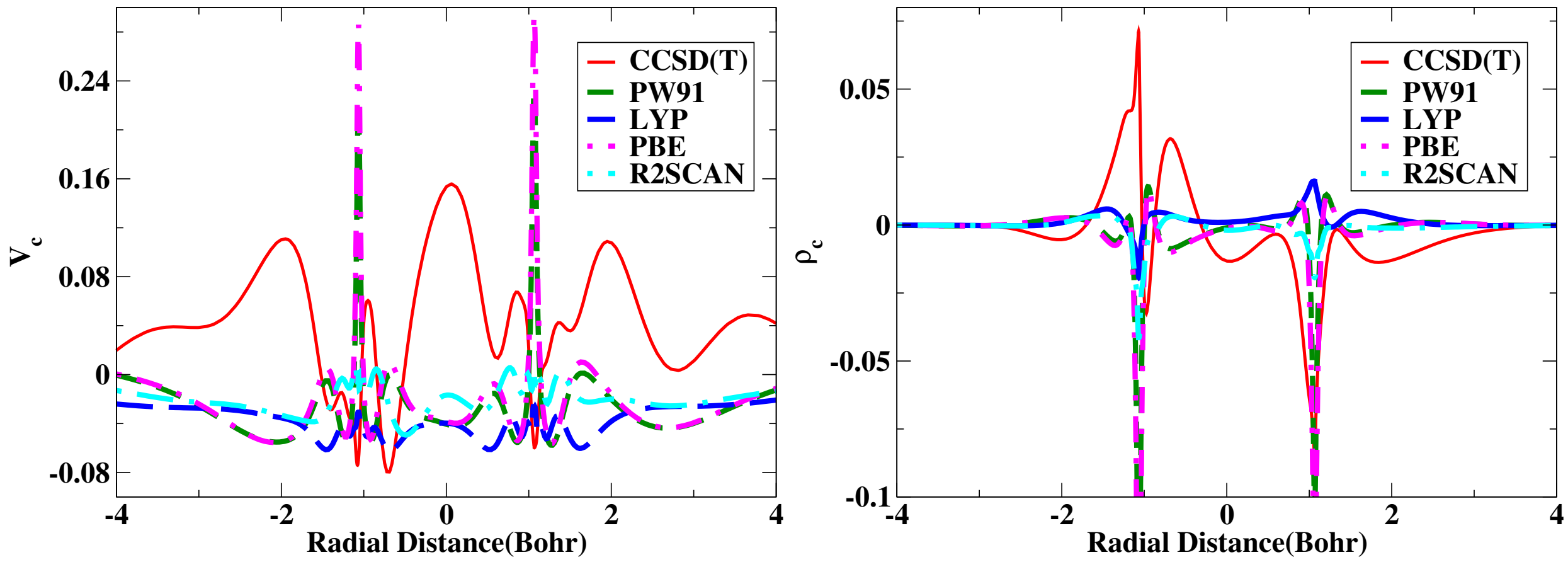

Figure S11: The correlation potentials (Eq. (9)) and correlated densities (Eq. (8)) computed from the self-consistent 100% HF+ $E_c^{\mathrm{DFA}}[\rho]$ calculations for CO molecule

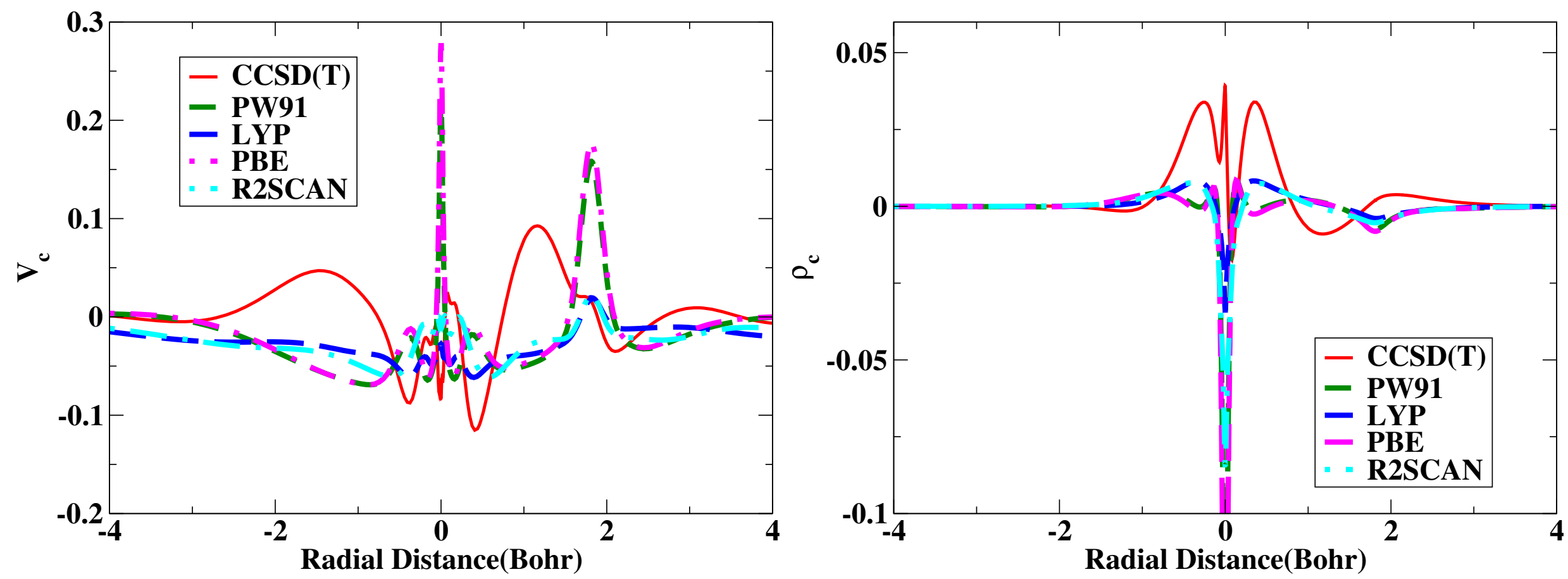

Figure S12: The correlation potentials (Eq. (9)) and correlated densities (Eq. (8)) computed from the self-consistent 100% HF+ $E_c^{\mathrm{DFA}}[\rho]$ calculations for the H_2O molecule

IV) UMK26 and UMK26x

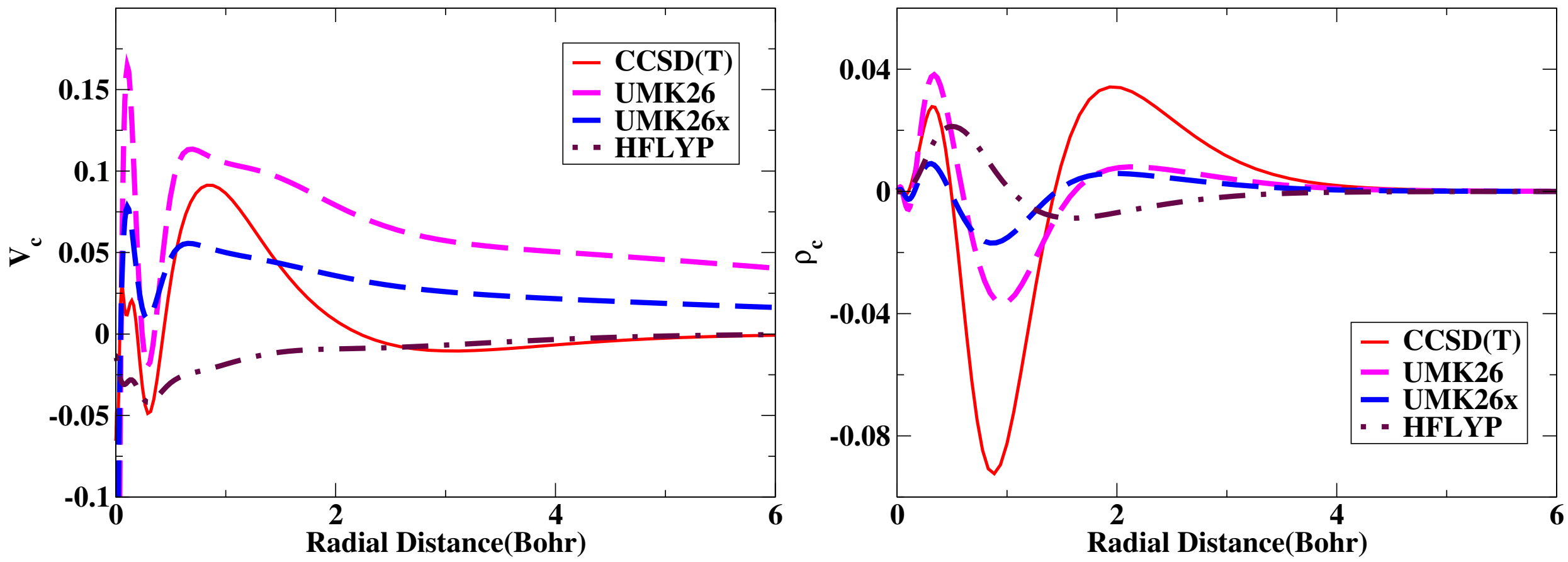

Figure S13: The correlation potentials and densities of UMK26 and UMK26x for Ne atom

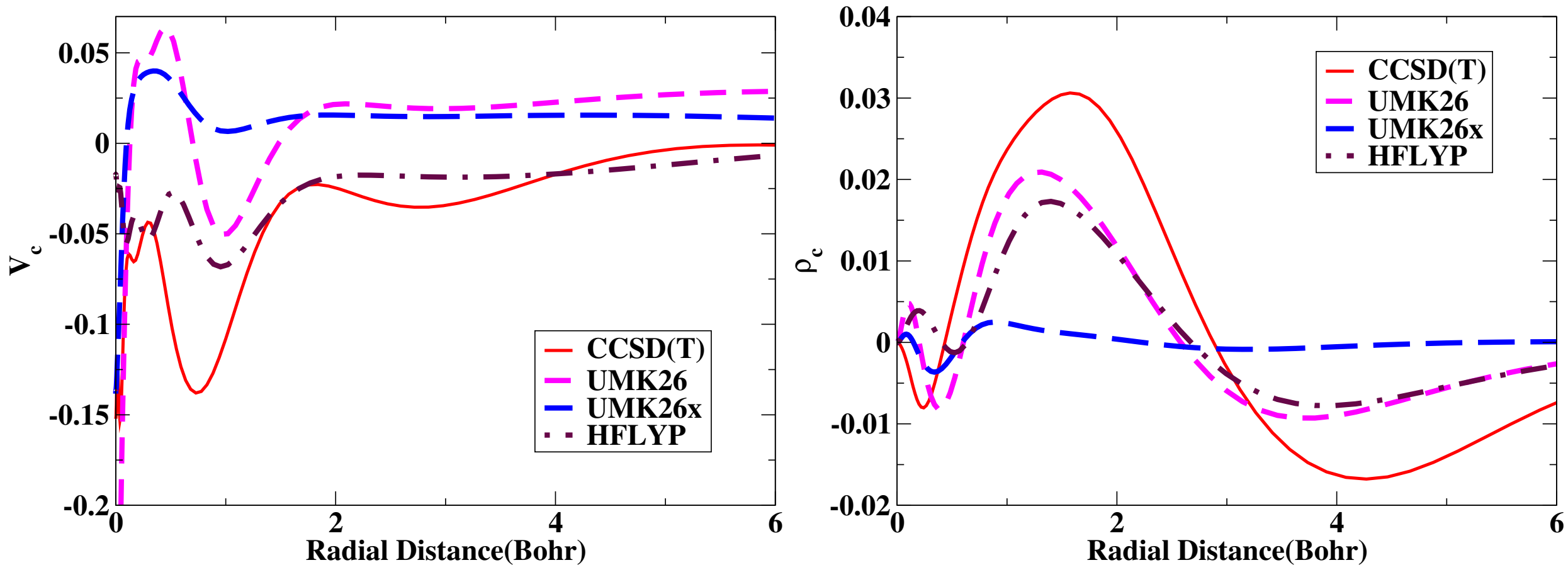

Figure S14: The correlation potentials and correlated densities of UMK26 and UMK26x for the Be atom

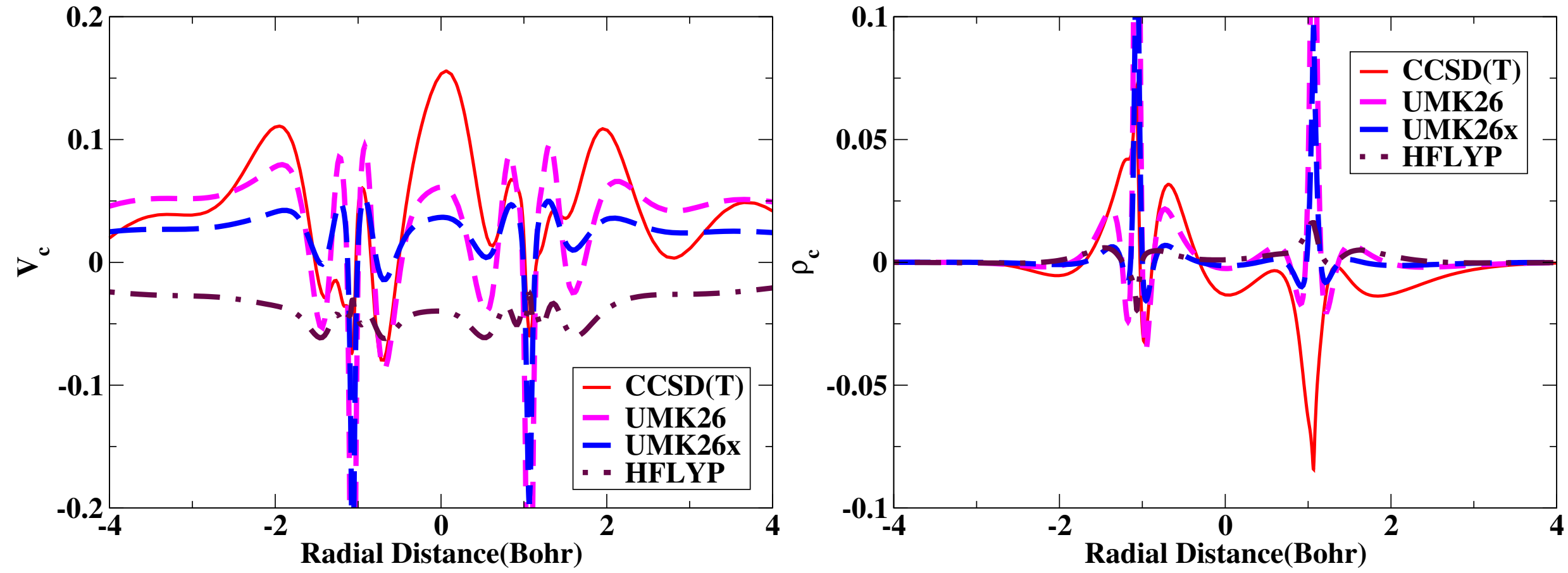

Figure S15: Correlation potential and correlated density for UMK26 and UMK26x for the CO molecule

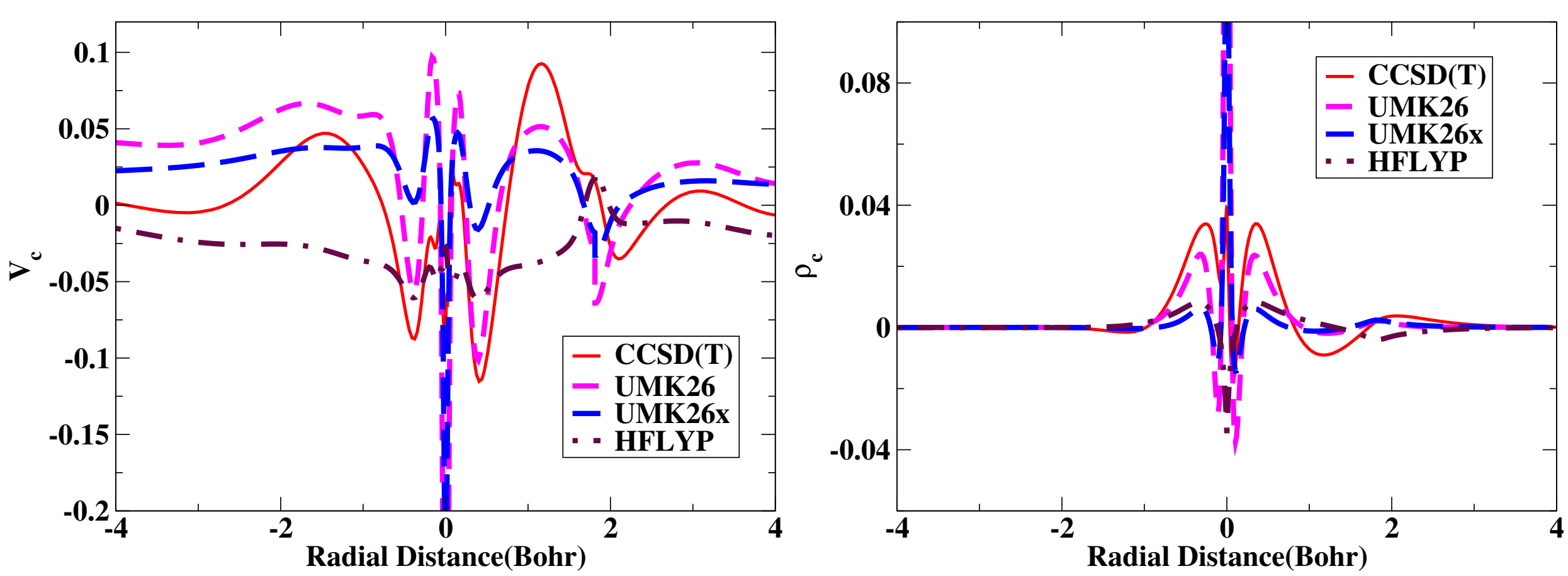

Figure S16: The correlation potentials and correlated densities of UMK26 and UMK26x for the H_2O molecule

Table S1: Vertical ionization potential (a.u.) computed for several systems with full and exchange-only parts of different hybrid functionals. All calculations have been performed in the uncontracted aug-cc-pVTZ basis set.

System	IP-EOM-CCSD	PBE0	$PBE0_x$	B3LYP	$B3LYP_x$	QTP17	$QTP17_x$	LC-ωPBE	LC-ωPBE_x
He	0.90156	0.66954	0.64252	0.66159	0.62246	0.80186	0.76157	0.77887	0.75210
H_2	0.60196	0.44014	0.41360	0.43361	0.40028	0.52559	0.49129	0.55544	0.52855
Be	0.34225	0.23863	0.21359	0.23222	0.20609	0.28319	0.25594	0.32767	0.30150
He_2	0.90151	0.66954	0.64252	0.66158	0.62245	0.80185	0.76156	0.77886	0.75209
Ne	0.78515	0.58841	0.55193	0.57562	0.53134	0.73886	0.69299	0.69047	0.65443
H_2O	0.46410	0.33397	0.30179	0.32467	0.28706	0.43773	0.39845	0.44402	0.41138
HF	0.59046	0.43548	0.40162	0.42469	0.38428	0.55997	0.51795	0.54341	0.50954
NH_3	0.38703	0.27239	0.24129	0.26397	0.22869	0.35829	0.32127	0.38149	0.34951
Mg	0.27789	0.1994	0.17549	0.19452	0.16946	0.2374	0.21112	0.27545	0.25036
CO	0.52234	0.39503	0.36363	0.38696	0.34991	0.48948	0.45109	0.50819	0.47644
N_2	0.57640	0.44869	0.41605	0.43992	0.40074	0.55805	0.51762	0.56159	0.52880
Ar	0.57617	0.44050	0.40395	0.42918	0.39053	0.53221	0.49238	0.55046	0.51350
C_2H_6	0.46270	0.34876	0.31866	0.34210	0.30649	0.42647	0.39000	0.45443	0.42407
HCl	0.46748	0.34954	0.31519	0.33948	0.30341	0.42836	0.39108	0.45726	0.42219
Ne_2	0.77988	0.58469	0.54787	0.57175	0.52714	0.73517	0.68907	0.68594	0.64950
Cl_2	0.42353	0.32079	0.28524	0.31112	0.27381	0.39769	0.35921	0.42501	0.38891
MAE		0.14531	0.17659	0.15421	0.19102	0.04676	0.08486	0.0403	0.07172
MARE [%]		25.941	31.982	27.608	34.553	8.496	15.694	05.748	11.839

Table S2: Correlation energies (in E_h) for several systems computed for B3LYP HYB functional using equation 7 (third column) and the correlation energy expression (fourth column) using self-consistent B3LYP. The last column presents the scaled energy difference. The MAE (in E_h) and MARE are calculated with respect to CCSD(T) reference data (second column). All calculations have been performed in the uncontracted aug-cc-pVQZ basis set.

Systems	CCSD(T)	Eq. (7)	$E_c^{\mathrm{B3LYP}}[\rho]$	$E_c^{\mathrm{XDiff}}[\rho]$
He	-0.0411819	-0.0546614	-0.0638648	0.0092034
H_2	-0.0404878	-0.0478979	-0.0556636	0.0077657
Be	-0.088351	-0.1023425	-0.1339201	0.0315776
He_2	-0.082365	-0.1093229	-0.1277298	0.0184069
Ne	-0.3621118	-0.4421277	-0.4901434	0.0480157
H_2O	-0.3509719	-0.4154888	-0.4387008	0.023212
HF	-0.3644942	-0.4388767	-0.4646248	0.0257481
NH_3	-0.3222876	-0.3772973	-0.4131716	0.0358743
Mg	-0.2189615	-0.4972266	-0.5882979	0.0910713
CO	-0.5025411	-0.5951173	-0.6256263	0.030509
N_2	-0.5173779	-0.6026224	-0.6238206	0.0211982
Ar	-0.4433619	-0.7598174	-0.9500528	0.1902354
C_2H_6	-0.534443	-0.6236359	-0.7188351	0.0951992
HCl	-0.4369698	-0.7509983	-0.9215902	0.1705919
Ne_2	-0.7244349	-0.8842862	-0.9806755	0.0963893
Cl_2	-0.8398284	-1.4675816	-1.7861369	0.3185553
MAE		0.144	0.22	
MARE[%]		37.27	58.55	

Table S3: Correlation energies (in E_h) for several systems computed for HYB functional LC-ωPBE using equation 7 (third column) and correlation energy expression (fourth column) of self-consistent LC-ωPBE. The last column presents the scaled energy difference. The MAE (in E_h) and MARE are calculated with respect to CCSD(T) reference data (second column). All calculations have been performed in the uncontracted aug-cc-pVQZ basis set.

Systems	CCSD(T)	Eq. (7)	$E_c^{\mathrm{LC-\omega PBE}}[\rho]$	$E_c^{\mathrm{XDiff}}[\rho]$
He	-0.0411819	-0.044564	-0.0411048	-0.0034592
H_2	-0.0404878	-0.0455631	-0.0431769	-0.0023862
Be	-0.088351	-0.0702062	-0.0857197	0.0155135
He_2	-0.082365	-0.0891291	-0.0822094	-0.0069197
Ne	-0.3621118	-0.380318	-0.34728	-0.033038
H_2O	-0.3509719	-0.3725685	-0.325567	-0.0470015
HF	-0.3644942	-0.3877069	-0.3355512	-0.0521557
NH_3	-0.3222876	-0.3423704	-0.3153051	-0.0270653
Mg	-0.2189615	-0.3877376	-0.4093355	0.0215979
CO	-0.5025411	-0.5160867	-0.4489112	-0.0671755
N_2	-0.5173779	-0.5276496	-0.4519499	-0.0756997
Ar	-0.4433619	-0.6137617	-0.7053523	0.0915906
C_2H_6	-0.534443	-0.5685066	-0.5603639	-0.0081426
HCl	-0.4369698	-0.6118993	-0.6873467	0.0754473
Ne_2	-0.7244349	-0.760682	-0.6946542	-0.0660278
Cl_2	-0.8398284	-1.1844388	-1.3282728	0.143834
MAE		0.067	0.090	
MARE[%]		17.87	20.33	

Table S4: Vertical excitation energies (eV) for charge-transfer excited states of different dimers, obtained in TDDFT calculations with the cc-pVDZ basis set and deviations with respect to the reference (J. Chem. Theory Comput. 2020, 16, 7, 4213).

System	Symm.	Ref.	PBE0	B3LYP	UMK26	QTP17	CAM-B3LYP	LC-ωPBE	LRC-ωPBEH	QTP00	QTP01	QTP02
NH_3···F_2	A_1	6.62	5.25	5.16	7.00	6.36	5.47	5.75	5.38	6.52	5.85	6.08
C_3H_6O···F_2	A''	5.90	1.76	1.32	6.32	5.02	2.82	3.89	2.51	5.47	4.04	4.59
$C_4H_4N_2$···F_2	B_2	6.33	2.16	1.71	6.77	5.42	3.34	4.59	3.08	5.29	4.54	5.18
C_3H_6O···CH_3NO_2	A	6.42	3.93	3.62	7.24	6.30	4.91	5.92	4.71	6.78	5.89	6.26
C_4H_5N···$C_4H_4N_2$[a]	B_1	5.27	2.77	2.50	5.39	4.68	4.16	5.62	4.38	5.55	5.39	5.61
	A_1	6.00	3.62	3.32	6.37	5.62	5.04	6.58	5.30	6.51	6.32	6.55
C_4H_5N···$C_4H_4N_2$[b]	A''	5.48	3.71	3.50	5.48	5.04	4.58	5.51	4.57	5.49	5.33	5.49
	A'	6.07	4.40	4.19	6.26	5.78	5.29	6.20	5.29	6.23	6.00	6.17
C_2F_4···C_2H_4 (5Å)	B_1	10.56	6.84	6.49	7.84	7.93	8.36	9.50	8.38	7.88	9.76	8.43
C_2F_4···C_2H_4 (3.5Å)	B_1	8.09	6.14	5.88	7.49	7.43	6.95	7.48	6.87	7.49	7.39	7.45
NH_3···OF_2	A'	7.09	2.90	2.45	7.18	6.01	4.13	5.45	3.77	6.58	5.35	5.87
NH_3···$C_4H_4N_2$	A'	7.53	4.10	4.06	5.60	5.59	4.28	4.38	4.20	5.54	4.43	5.47
	A'	7.83	5.26	5.05	6.33	6.56	5.40	5.38	5.40	5.95	5.44	5.51
MAD			2.80	3.07	0.74	0.88	1.88	1.16	1.95	0.81	1.10	0.96
MAX			4.19	4.64	2.72	2.63	3.25	3.15	3.39	2.68	3.10	2.32
MSD			−2.80	−3.07	−0.30	−0.88	−1.88	−0.99	−1.95	−0.61	−1.04	−0.81

[a] H-bonded.

[b] Stacked.

Table S5: Intramolecular charge-transfer excitations (eV) w.r.t theoretical best estimates (TBE) from Loos et al (J. Chem. Theory Comput. 2021, 17, 6, 3666–3686). The basis set was cc-pVTZ.

System	Symm.	TBE	PBE0	B3LYP	QTP17	UMK26	UMK26x	CAM-B3LYP	LC-ωPBE	LRC-ωPBEH	QTP00	QTP01	QTP02
Aminobenzonitrile	$2A_1$	5.26	5.10	5.02	5.38	5.40	5.70	5.21	5.34	5.23	5.38	5.32	5.35
Aniline	$2A_1$	5.87	5.70	5.61	5.89	5.87	6.10	5.77	5.87	5.80	5.87	5.84	5.86
Azulene	$2A_1$	3.89	3.71	3.63	3.82	3.76	3.99	3.75	3.84	3.76	3.78	3.80	3.81
	$2B_2$	4.55	5.86	4.66	5.00	5.04	5.47	4.79	4.88	5.99	4.98	4.87	4.91
Benzonitrile	$1A_2$	7.10	6.31	6.21	6.64	6.63	6.88	6.62	6.87	6.60	6.73	6.79	6.81
Benzothiadiazole	$1B_2$	4.37	3.94	3.83	4.26	4.57	4.64	4.15	4.43	4.18	4.32	4.34	4.37
Dimethylaminobenzonitrile	$2A_1$	4.94	4.80	4.72	5.16	4.77	5.55	4.97	5.11	4.97	5.19	4.85	5.14
Dimethylaniline	$1B_1$	4.47	4.57	4.48	5.07	5.21	5.53	4.74	4.88	4.72	5.10	4.89	4.96
	$2A_1$	5.54	5.40	5.32	5.71	5.74	6.00	5.53	5.65	5.54	5.72	5.64	5.68
Dipeptide	$7A''$	8.15	7.24	7.07	8.48	8.51	7.98	7.83	8.67	7.95	8.28	8.24	8.38
β-dipeptide	$7A'$	8.51	6.77	6.51	7.80	8.04	8.53	7.28	7.60	7.96	7.84	7.51	7.62
	$10A''$	8.90	6.90	6.84	8.64	9.11	9.41	7.92	8.87	8.12	8.89	8.45	8.59
HCl	1Π	8.10	7.93	7.68	8.25	8.34	8.45	7.82	8.23	7.99	8.20	7.94	8.01
Nitroaniline	$2A_1$	4.57	4.18	4.04	4.78	4.95	5.35	4.47	4.79	4.51	4.88	4.74	4.81
Nitrobenzene	$2A_1$	5.57	5.00	4.83	5.45	5.52	5.83	5.22	5.55	5.27	5.51	5.45	5.51
Nitrodimethylaniline	$2A_1$	4.28	3.92	3.78	4.56	4.75	5.17	4.25	4.58	4.30	4.69	4.53	4.61
Nitropyridine-N-Oxide	$2A_1$	4.24	4.04	3.92	4.43	4.47	4.91	4.26	4.45	4.32	4.46	4.42	4.44
N-Phenylpyrrole	$2B_2$	5.53	4.82	4.66	5.35	5.45	5.72	5.08	5.24	5.10	5.38	5.23	5.28
	$3A_1$	6.04	5.17	5.01	6.46	6.69	7.01	6.00	6.70	6.06	6.68	6.64	6.67
Phthalazine	$1A_2$	3.93	3.64	3.50	4.71	5.09	5.42	4.05	4.26	3.94	4.84	4.37	4.51
	$1B_1$	4.34	4.05	4.64	4.83	5.10	5.48	4.39	4.59	4.32	4.95	4.66	4.76
Quinoxaline	$1B_2$	4.74	3.58	4.15	4.21	4.84	5.17	4.57	3.95	4.57	4.30	4.82	4.11
	$3A_1$	5.75	5.89	4.47	6.26	6.36	6.79	6.00	6.15	4.61	6.28	6.13	6.19
	$2B_1$	6.33	5.92	5.76	6.46	6.54	7.00	6.28	7.02	6.40	6.53	7.09	6.52
Twisted-Dimethylaniline	$1A_2$	4.17	3.38	3.23	4.73	5.31	5.86	3.98	4.40	3.90	5.02	4.48	4.66
	$1B_1$	4.84	4.10	3.91	5.52	5.59	5.84	4.78	5.34	4.64	5.50	5.36	5.41
Twisted-N-Phenylpyrrole	$2B_2$	5.73	4.57	4.37	5.73	5.81	6.05	5.38	5.56	5.41	5.73	5.58	5.62
	$2A_1$	5.82	4.68	4.46	6.05	6.10	6.30	5.62	6.30	5.68	6.16	6.21	6.23
	$1A_2$	6.04	5.20	5.00	6.51	6.62	7.27	5.94	6.60	5.98	6.87	6.56	6.71
	$1B_1$	6.28	5.34	5.11	6.85	6.45	7.92	6.19	7.04	6.24	7.27	6.95	7.12
MAD			0.64	0.74	0.33	0.38	0.67	0.23	0.33	0.25	0.37	0.31	0.34
MAX			2.00	2.07	0.78	1.16	1.69	1.23	0.91	1.44	0.99	1.00	0.89
MSD			−0.54	−0.71	0.17	0.29	0.65	−0.17	0.16	−0.13	0.25	0.13	0.16

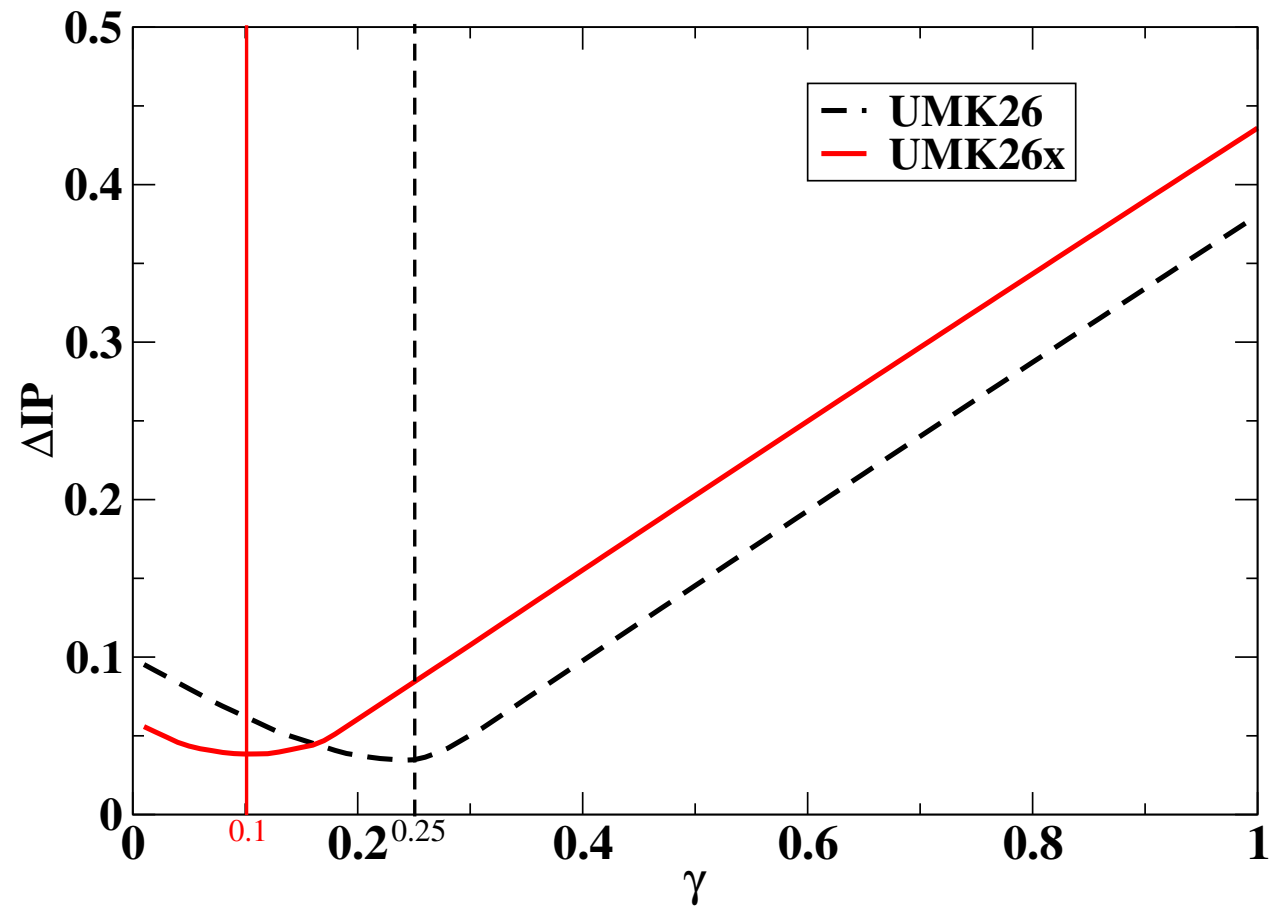

Figure S17: ΔIP error with respect to the γ parameter for the optimization of UMK26 and UMK26x BLYP-based functional. The vertical lines indicate the optimal values obtained for UMK26 and UMK26x DFAs.

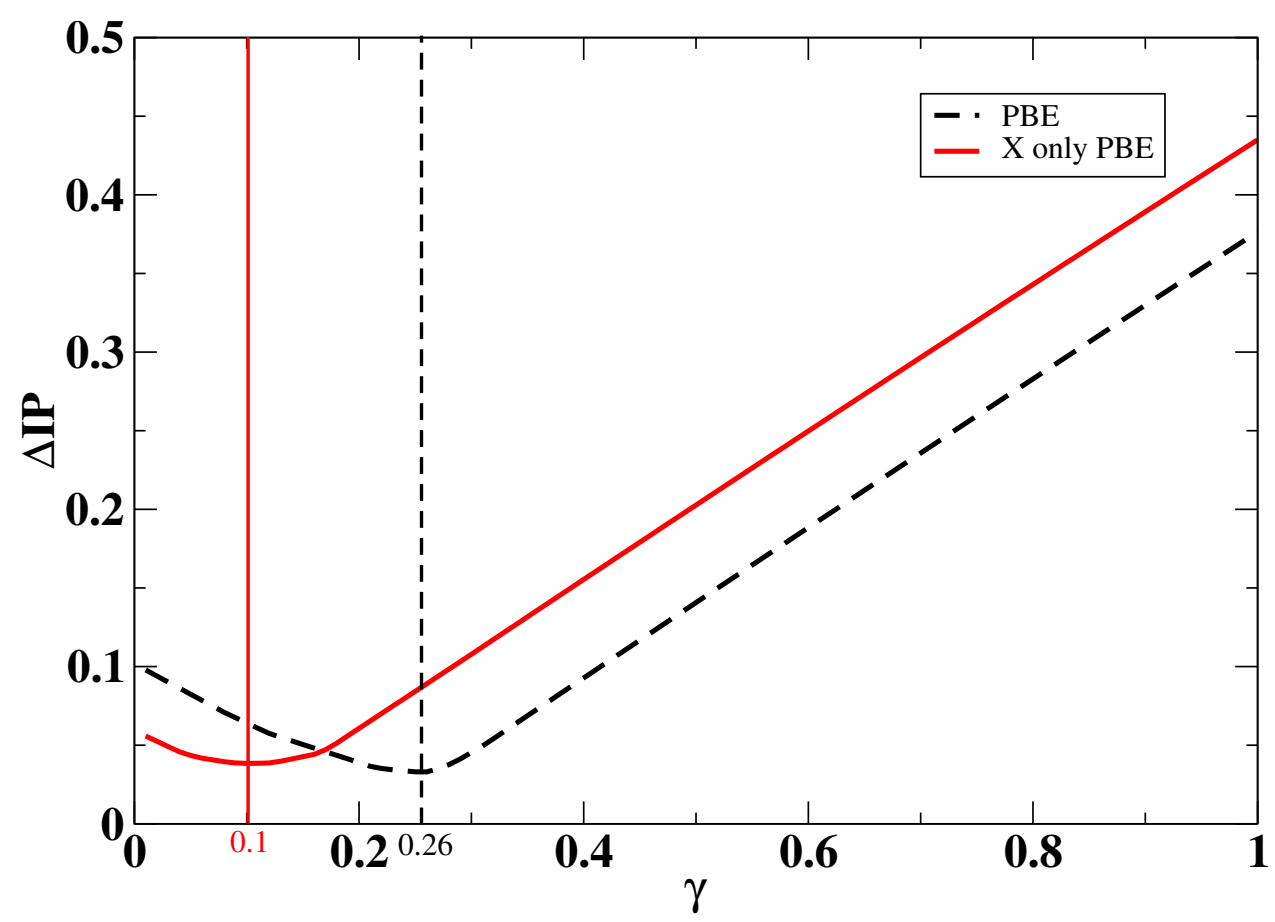

Figure S18: ΔIP error with respect to the γ parameter for the optimization of γ in Eq. 10 with PBE exchange and correlation expressions. The dashed black curve represents the full hybrid functional constructed as $E_{xc}^{\mathrm{HYB}}[\rho] = E_x^{\mathrm{EXX}} + \gamma\big(E_x^{\mathrm{PBE}}[\rho] - E_x^{\mathrm{EXX}}\big) + E_c^{\mathrm{PBE}}[\rho]\big)$ and the red curve corresponds to exchange only construction as $E_{xc}^{\mathrm{HYB}}[\rho] = E_x^{\mathrm{EXX}} + \gamma\big(E_x^{\mathrm{PBE}}[\rho] - E_x^{\mathrm{EXX}}\big)$. The vertical lines indicate the optimal values obtained for both cases.
